# Wearable perovskite solar cells by aligned liquid crystal elastomers

**DOI:** 10.1038/s41467-023-36938-7

**Published:** 2023-03-02

**Authors:** Zengqi Huang, Lin Li, Tingqing Wu, Tangyue Xue, Wei Sun, Qi Pan, Huadong Wang, Hongfei Xie, Jimei Chi, Teng Han, Xiaotian Hu, Meng Su, Yiwang Chen, Yanlin Song

**Affiliations:** 1 Key Laboratory of Green Printing, Institute of Chemistry, Chinese Academy of Sciences (ICCAS), Beijing Engineering Research Center of Nanomaterials for Green Printing Technology, Beijing National Laboratory of Molecular Sciences (BNLMS), Beijing, 100190 P. R. China; 2 Key Laboratory of Fluorine and Silicon for Energy Materials and Chemistry of Ministry of Education, College of Chemistry and Chemical Engineering, Jiangxi Normal University, 99 Ziyang Avenue, Nanchang, 330022 P. R. China; 3 Research Center for Green Printing Nanophotonic Materials, School of Materials Science and Engineering, Suzhou University of Science and Technology, Suzhou, 215009 P. R. China; 4 University of Chinese Academy of Sciences, Beijing, 100149 P. R. China; 5 School of Materials Science and Engineering, Zhengzhou University, Zhengzhou, 450001 P. R. China; 6 Institute of Software, Chinese Academy of Sciences (ISCAS), Beijing, 100190 P. R. China; 7 College of Chemistry, Nanchang University, 999 Xuefu Avenue, Nanchang, 330031 P. R. China

**Keywords:** Solar cells, Liquid crystals

## Abstract

In a flexible perovskite solar cell, the bottom interface between perovskite and the electron-transporting layer is critical in determining its efficiency and reliability. High defect concentrations and crystalline film fracturing at the bottom interface substantially reduce the efficiency and operational stability. In this work, a liquid crystal elastomer interlayer is intercalated into a flexible device with the charge transfer channel toughened by the aligned mesogenic assembly. The molecular ordering is instantly locked upon photopolymerization of liquid crystalline diacrylate monomers and dithiol-terminated oligomers. The optimized charge collection and the minimized charge recombination at the interface boost the efficiency up to 23.26% and 22.10% for rigid and flexible devices, respectively. The liquid crystal elastomer-induced suppression of phase segregation endows the unencapsulated device maintaining >80% of the initial efficiency for 1570 h. Moreover, the aligned elastomer interlayer preserves the configuration integrity with remarkable repeatability and mechanical robustness, which enables the flexible device to retain 86% of its original efficiency after 5000 bending cycles. The flexible solar cell chips are further integrated into a wearable haptic device with microneedle-based arrays of sensors to demonstrate a pain sensation system in virtual reality.

## Abstract (web summary)

The bottom interface between perovskite and electron-transporting layer is critical for realizing efficient flexible perovskite solar cells. Here, the authors intercalate liquid crystal elastomer interlayer for aligned mesogenic assembly and achieve maximum efficiency of 23.26% with *T*_80_ over 1570 h.

## Introduction

Perovskite solar cells (PSCs) have attracted considerable attention for next-generation photovoltaic applications, such as building-integrated photovoltaics, intelligent vehicles, and wearable electronics^1–3^, due to high power conversion efficiency (PCE, >25%^4^), lightweight, flexibility, and low-cost benefiting from the favorable optoelectronic properties of hybrid organic-inorganic halide perovskites and low processing temperature^5–8^. However, their commercialization has been impeded by undesirable reliability with respect to long-term operational stability and mechanical endurance^9^, which mostly originates from defects that aggregate at layer interfaces in typical PSCs^10–13^. The recombination at interfaces increases as the interfacial area between the perovskite and charge transport layers extends, especially in a planar configuration, which requires more efficient charge extraction and transfer with low interfacial recombination loss^13,14^. Notably, the charge accumulation due to the imperfect electronic contact at the electron-transport layer (ETL)/perovskite interface (bottom interface) is more significant than that at the perovskite/hole-transport layer (HTL) interface. In essence, the bottom interface generally contains a high concentration of defects, specifically deep-level defects, which leads to hysteresis and irreversible degradation of PSCs^10,15,16^.

With many strategies attempted to reduce interfacial defects at the bottom interface, like adding interfacial layers^17,18^, interpenetrating interfaces^13,14,19^, scaffolding^20–22^, and introducing additives^23–25^, introduction of interfacial self-assembled monolayers (SAMs) turns out to be a simple and effective way to minimize charge-transport losses and suppress charge recombination^26,27^. However, unavoidable defects in these monolayers stemming from the flexible substrate roughness and film depositions can directly undermine the performance and stability of flexible PSCs^28^. To solve this problem, silane coupling agents have been utilized to form a cross-linked SAM via a silanization process^29,30^, which could not only reduce hysteresis and improve the operational stability, but also preserve the mechanical tolerance for flexible devices^30,31^. However, a trade-off between passivation quality (open-circle voltage, *V*_oc_) and series resistance (fill factor, FF) originating from the heterogeneous contact of silanes is still unavoidable. Thus, strategies with effective surface passivation ability and good carrier conductivity are in urgent demand to overcome this trade-off to enhance the performance and reliability of PSCs.

Liquid crystalline elastomers (LCEs) are lightly cross-linked polymer networks featured with anisotropy from self-assembled mesogens and elasticity from polymers with a variety of applications in soft robotics, photonic displays, biomedical engineering, and flexible electronics^32^. The degree of the order of a LCE network highly depends on the mesogenic alignment within the elastomeric network and can be manipulated via various alignment methods. LCE collaborating with functional inorganic materials synergically make it possible to give birth to hybrid composites with improved diverse properties, such as mechanical, electrical, optical, and actuation properties as well as tailorable responsiveness, which consequently broadens the application scope of LCE^33^. For instance, composites of planar nematic LCE and carbon nanotubes (CNTs) could present a high anisotropy of surface resistivity^34^, which provides a possibility for the application of LCEs in integrating flexible solar cells.

Here, we fabricate PSCs with a well-aligned interlayer formed by thiol-terminated LCE that acts as a toughening charge transfer channel between SnO_2_ and perovskite thin films. The molecular ordering is instantly locked upon photopolymerization of liquid crystalline diacrylate monomers and dithiol-terminated oligomers. The aligned interlayer can maintain excellent charge collection and minimize charge recombination at the SnO_2_/perovskite interface making the resulted rigid PSC device deliver a PCE up to 23.26% with a *V*_oc_ of 1.17 V and an FF of 0.803 as well as reduced hysteresis. Moreover, the LCE interlayer can preserve the configurational integrity by toughening the ETL/perovskite interface and suppressing the perovskite phase segregation under continuous illumination. The unencapsulated device shows a *T*_80_ lifetime (the time over which the device efficiency reduces to 80% of the initial value) of over 1570 h under flowing N_2_. Interestingly, flexible PSCs modified with the aligned LCE interlayer also display high PCE values up to 22.10% with a *V*_oc_ of 1.15 V and an FF of 0.780. These devices all demonstrate remarkable repeatability and reliability as well as excellent mechanical endurance, which retains 86% of the initial efficiency after 5000 bending cycles. Additionally, the reliable solar cell chips are further applied to successfully power a wearable haptic device for pain sensation in virtual reality.

## Results

### Synthesis and characterization of LCE interlayer

**Fig. 1:**
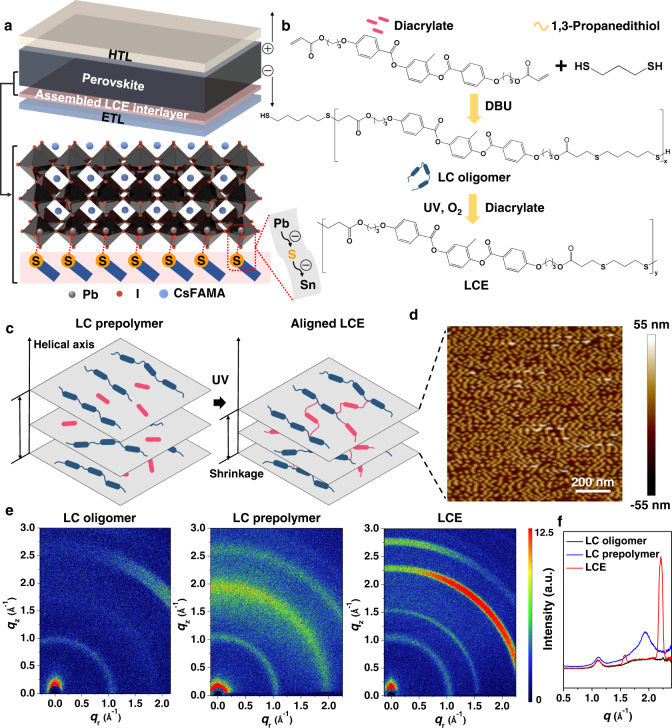
Synthesis and characterization of LCE. **a** Schematic illustration of the LCE-assisted PSC with an assembled LCE interlayer intercalated between the perovskite absorber and the ETL layer. **b** Synthetic route of LCE. **c** Schematic illustration of aligned-LCE assembled via photopolymerization from randomly orientated LC prepolymer. **d** AFM height image of LCE on ITO glass. **e** GIWAXS patterns, and **f** the corresponding intensity profiles as a function of the scattering vector *q* of the LC oligomer, LC prepolymer, and LCE fabricated on ITO glass, respectively.

For a typical multilayer-structured PSC with HTL, ETL, and perovskite layers, an LCE interlayer is desirably intercalated between the perovskite absorber and the ETL layer (Fig. 1a). The growth of perovskite crystals is anchored by the aligned LCE interlayer. LC oligomer with terminated dithiols is firstly prepared in the lab via the prepolymerization reaction between a diacrylate monomer (RM257) and a dithiol (1,3-propanedithiol) at a molar ratio of 1:2 with a basic catalysis. Then LCE can be obtained by an oxygen-mediated thiol-acrylate click reaction upon UV exposure on the LC prepolymer containing the lab-made LC oligomer and the diacrylate monomer (RM257) at a molar ratio of 1:1 (Fig. 1b)^35^. The synthesized LCE is confirmed by Fourier-transform infrared spectroscopy (FTIR), which indicated the conversion of acrylate based on the disappearance of peaks at 821 cm^−1^ and 1641 cm^−1^ (C=C stretching), respectively, under UV irradiation (Supplementary Fig. 1). The resulted LCE thin film shows birefringence when observed by polarized optical microscopy (POM) under crossed polarizers indicating a nematic LC phase (Supplementary Fig. 2)^36^. A cross-linked LCE network with well-aligned mesogens can be achieved from the randomly orientated LC prepolymer with the elimination of solvent and the exposure of UV (Fig. 1c). Morphologies of LC oligomer, LC prepolymer and LCE films on ITO glass are investigated, respectively. Compared with the morphologies of LC oligomer and LC prepolymer films (Supplementary Fig. 3), the LCE film exhibits a periodic morphology due to the instant locking of molecular ordering (Fig. 1d)^35,36^. We further use grazing incidence wide-angle X-ray scattering (GIWAXS) to study the molecular stacking in these three LC films^37,38^. The 2D GIWAXS patterns and the reduced scattering intensities versus X-ray momentum transfer *q* (defined as *q* = 4*π*sin*θ*/*λ*, in which 2*θ* is the scattering angle and *λ* is the wavelength of the incident X-rays) are shown in Fig. 1e and Fig. 1f, respectively. It displays obvious scattering peaks owing to variations in the ordering of the film microstructures. In comparison to the scattering peak observed for the LC oligomer film at *q* of ~1.1 Å, the LC prepolymer film presents an additional wide scattering peak at *q* of ~1.9 Å, which corresponds to the disordered diacrylate monomer. After UV irradiation for 5 min, the scattering peak at *q* of ~1.9 Å disappears along with the appearance of two major peaks at *q* of ~1.6 Å and ~2.2 Å, respectively, which suggests the formation of the aligned LCE film with molecular ordering instantly locked by UV-induced photopolymerization. A ~9.6 nm thin film is constructed after this photopolymerization (Supplementary Fig. 4).

### Perovskite crystal growth mechanism

The effects of LCE films on the growth of perovskite crystals are further evaluated. X-ray diffraction (XRD) measurements reveal the crystalline orientation and quality of the perovskite films (Supplementary Fig. 5). Basically, the diffractograms of all the samples show the same trigonal perovskite crystal structure. The full-width-at-half-maximum (FWHM) of the (001) perovskite lattice reduces from 0.167 for the perovskite sample on SnO_2_ to 0.148 for the perovskite on SnO_2_/LCE sample as LCE interlayer is inserted, which proves the enhanced crystallinity of the perovskite film. Additionally, the standard diffraction peak intensity of PbI_2_ at 12.6° is obviously lowered, which indicates that the content of PbI_2_ in the perovskite surface is reduced and accordingly charge transfer is promoted. Moreover, the XRD peak intensity ratio of (001) versus (012) is boosted for the perovskite film fabricated on SnO_2_/LCE (Supplementary Table 1). These results demonstrate the preferential orientation along the (001) direction for perovskite crystal growth on the LCE interlayer^39^, which is further confirmed by the strong out-of-plane signal in z axis in the GIWAXS 2D patterns (Supplementary Fig. 6). From scanning electron microscopy (SEM) images of the perovskite film on pristine SnO_2_ (Supplementary Fig. 7), significant pin holes and interface voids are observed. In addition, some bright crystals are lying on both top and bottom sides of the perovskite film, which can be ascribed to the presence of PbI_2_. By contrast, the perovskite film fabricated on SnO_2_/LCE exhibits uniform morphology and compact perovskite crystals along the vertical direction.

According to the classical nucleation theory of thin films^40,41^, the Gibbs free energy for heterogeneous nucleation in the formation of nuclei can be expressed as the following equation:

1 $${\Delta G}_{{{{{{\rm{heterogeneous}}}}}}}={\Delta G}_{{{{{{\rm{homogeneous}}}}}}}\times f(\theta )$$

wherein $f\left(\theta \right)=\frac{1}{4}\left\{2+{{\cos }}\theta \right\}{\left\{1-{{\cos }}\theta \right\}}^{2}$, and *θ* is the contact angle (the magnitude of *θ* varies in the range of [0, π/2])^42^. The smaller contact angle can result in a smaller *f*(*θ*) and reduced Gibbs free energy, thereby assisting the nucleation process for the heterogeneous nucleation. Therefore, the surface properties of SnO_2_ and SnO_2_/LCE are carried out to understand the influence of aligned LCE on the nucleation and crystal growth of perovskites. As shown in Supplementary Fig. 8, the SnO_2_/LCE shows a smaller contact angle (13.4°) than SnO_2_ (19.6°). The small contact angle of this SnO_2_/LCE interface provides high surface energy and nucleation density, which promote the film densification process, as observed in the SEM images.

### Charge transfer and recombination mechanism

**Fig. 2:**
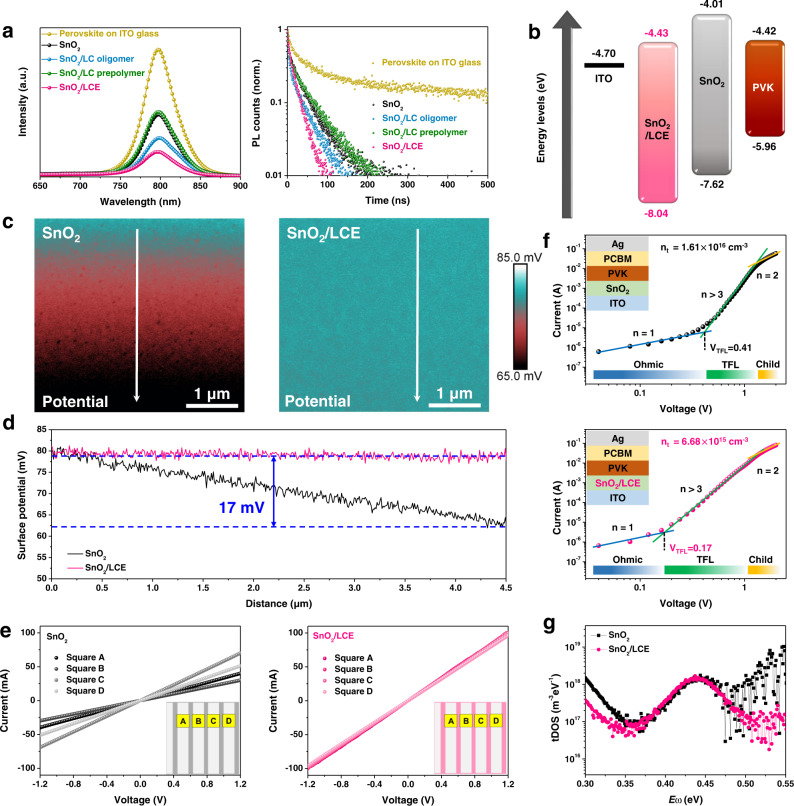
Charge transfer and recombination characterization for SnO_2_ and SnO_2_/LCE. **a** PL and time-resolved PL spectra of perovskite films deposited on various substrates. **b** Schematic illustration of the energy levels. **c** KPFM images of SnO_2_ films without (left) and with (right) LCE. **d** Line profiles taken at the position marked by white arrows in the KPFM images. **e** *I–V* curves of devices based on the structure of ITO/ETL/Ag without (left) and with (right) LCE interlayer. The inset shows the selected squares. **f** *I*–*V* curves of the electron-only devices fabricated without (up) and with (down) LCE interlayer. **g** *t*DOS in perovskite solar cells with or without LCE interlayer.

Photoluminescence (PL) and time-resolved PL spectra for CsFAMA perovskite films fabricated on various substrates are investigated (Fig. 2a). The perovskite film on pristine SnO_2_ exhibits substantially lower PL intensity than that on ITO glass, which indicates the presence of SnO_2_ causes an enhanced interfacial non-radiative recombination and/or attenuated radiative recombination through non-radiative recombination channels (charge transfer to ETLs) of photogenerated electron holes^13^. The interfacial non-radiative recombination of the perovskite film on SnO_2_/LC prepolymer is close to that on SnO_2_ because of their similar interfacial contacts. Interestingly, the perovskite film on SnO_2_/LCE exhibits the lowest photoluminescence intensity, which implies the presence of LCE interlayer leads to the most effective charge carrier transfer. The charge carrier dynamics are further explored by time-resolved PL to disclose the origin of the reduced charge recombination. The fast (*τ*_1_) and slow (*τ*_2_) decay components of the bi-exponential decay function y = *A*_1_exp(−*t*/*τ*_1_) + *A*_2_exp(−*t*/*τ*_2_) + y_0_ are analyzed, where *A*_n_ (*n* = 1, 2) and *t* are the amplitude components and the time constant, respectively. The fast-decay component (*τ*_1_) results from the trap-assisted charge recombination of free-carriers transporting to the electrode while the slow-decay component (*τ*_2_) derives from the radiative recombination in the bulk perovskite^43,44^. As shown in Supplementary Table 2, the fast decay lifetime is the shortest for the SnO_2_/LCE supported perovskite film and the weight fraction of this lifetime is more pronounced. These results demonstrate that the molecular ordering interlayer can not only reduce the non-radiative interfacial recombination but also promote the photogenerated charge transfer to the electrode. Moreover, the conduction band minimum (CBM) and the valence band maximum (VBM) of SnO_2_/LCE are −4.43 eV and −8.04 eV, respectively (Fig. 2b and Supplementary Fig. 9). Compared with the pristine SnO_2_, the relative downshift of the CBM in the SnO_2_/LCE film facilitates more efficient electron injection from the perovskite into the ETL. Atomic force microscopy (AFM) combined with Kelvin probe force microscopy (KPFM) is further applied to elucidate the effect of LCE on the film surfaces. The topographic spatial map (Supplementary Fig. 10) and the corresponding surface potentials (Fig. 2c) of SnO_2_/LCE reveal distinguished discrepancies with respect to film morphologies and potentials between the SnO_2_ and the SnO_2_/LCE films. In essence, the pristine SnO_2_ film exhibits a surface potential difference of ~17 mV in the selected vertical area (linear section from KPFM images) while a negligible surface potential change is observed for the SnO_2_/LCE film (Fig. 2d), which demonstrates the existence of uniform charge transfer channels. To investigate the conductivity of the uniform charge transfer channel, we perform the *I–V* plots of devices based on the structure of ITO/ETL/Ag (Fig. 2e). The SnO_2_/LCE-based device shows identical *I–V* curves from each of four selected squares while non-superimposable curves are obtained for the pristine SnO_2_ based devices. In addition, the conductivity of the devices with 3-aminopropyl tri-ethoxysilane (APTES) and LC oligomer are measured, respectively, to highlight the efficient charge transfer in the LCE layer (Supplementary Fig. 11). The space charge limited current (SCLC) analysis of the electron-only devices displays that the trap-filling limit voltage (*V*_TFL_) of the SnO_2_/LCE-based device (*V*_TFL_ = 0.17 v) is much lower than that of the SnO_2_ based device (*V*_TFL_ = 0.41 v), leading to a decreased trap density by an order of magnitude from 1.61 × 10^16 ^cm^−3^ to 6.68 × 10^15 ^cm^−3^ (Fig. 2f). The calculations of the trap density are detailed in Supplementary Note 1. The trap density of states (*t*DOS) of the as-fabricated devices is also deduced from the thermal admittance spectroscopy. As shown in Fig. 2g, the *t*DOS, as a function of the defect energy, demonstrates a reduction in trap states for the SnO_2_/LCE-based device compared with the SnO_2_ based device. On account of the same perovskite composition and film architecture, the reduced trap density can be mainly ascribed to the ordered LCE interlayer that results in robust interfacial contact. The reduced level of defects is further evidenced by conducting X-ray photoelectron spectroscopy (XPS) analysis near the bottom of the perovskite film (Supplementary Fig. 12). The exposure of bottom perovskite is achieved by ion beam thinning. Compared to the perovskite film on SnO_2_, the binding energy for the SnO_2_/LCE-based perovskite film with respect to Pb 4*f*_7/2_ and Pb 4*f*_5/2_ is observed to be decreased by 0.2 eV, indicating the oxidation state of lead is lowered due to the electron donation from sulfur atom^45^, which is consistent with the shifted stretching vibration of –SH in FTIR spectrum (Supplementary Fig. 13). Consequently, this result suggests that molecular ordered LCE interlayer can greatly reduce the density of coordinatively unsaturated Pb^2+^ sites at the bottom surface.

### Performance and operational stability of PSCs in rigid versions

**Fig. 3:**
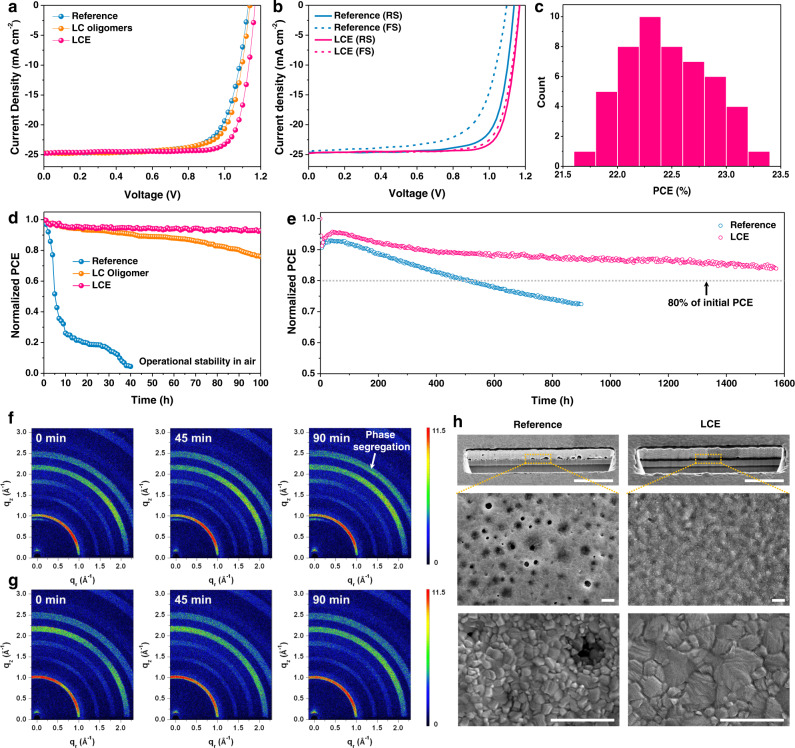
Photovoltaic performance and operational stability of PSCs. **a** *J–V* curves of the champion PSC devices based on reference, LC oligomer, and LCE. **b** *J–V* curves of PSCs measured in reverse and forward modes. **c** The efficiency distribution of 50 devices based on LCE. **d** Operational stability of the unencapsulated PSC devices based on reference, LC oligomers, and LCE in ambient air (40% RH). **e** Continuous operational stability of the unencapsulated PSC devices based on reference and LCE under flowing N_2_. GIWAXS patterns collected under vacuum during the operation of **f** the reference PSC and **g** LCE-based PSC for 0, 45 min, and 90 min. **h** Cross-sectional and bottom side-view SEM images of the corresponding PSCs collected after operational stability test. Scale bar: 1 μm.

PSCs with different interlayers based on a typical planar n-i-p architecture are fabricated and tested. Compared with the reference PSC (devices based on SnO_2_), the PSC fabricated with SnO_2_/LC oligomer shows negligible performance enhancement (Fig. 3a and Supplementary Table 3), which is mainly due to that the disorder of LC oligomer cannot provide an effective charge transfer channel between the perovskite/ETL interface. Notably, the current density–voltage (*J–V*) characteristics show that the LCE-based PSC can deliver a PCE of 23.26% with an elevated *V*_oc_ of 1.17 V, a short-circuit current density (*J*_sc_) of 24.79 mA cm^−2^, and an FF of 0.80. The optimized concentration (0.1 mg mL^−1^) of LCE in PSCs is shown in Supplementary Fig. 14. The *J*_sc_ values are consistent with those integrated by the external quantum efficiency (EQE) spectra (24.60 mA cm^−2^) (Supplementary Fig. 15). The optical bandgap and the CBM of the CsFAMA PSC are determined to be 1.54 eV (Supplementary Fig. 16) and −4.42 eV (Supplementary Fig. 17), respectively. Compared with the CBM of pristine SnO_2_ (−4.01 eV), the relative drop of the CBM in the SnO_2_/LCE (−4.43 eV) can facilitate a more efficient electron injection from perovskite into the ETL, which demonstrates a less charge accumulation at the ordered bottom interface. As a result, increased *V*_oc_ and lower hysteresis index (0.030) are observed for the LCE-based PSC (Fig. 3b). The reduced hysteresis index and suppressed charge accumulation indicate an inhibition of the migration of ionic defects in perovskite phase, which is important for boosting the device performance and stability. The stabilized output power of the LCE-based device is presented in Supplementary Fig. 18, which delivers a stabilized PCE of 22.38% at maximum power point. The LCE-based PSCs turn out to display excellent reproducibility based on 50 trials with PCE values summarized in Fig. 3c; for example, 88% of the PSCs are with PCEs more than 22%. The enhancement in PCE values for the LCE-based PSCs largely results from the increased *V*_oc_ and FF, which is ascribed to the lower trap density and the efficient electron injection benefiting from such uniform charge transfer channel. To further unravel the underlying charge carrier recombination and transfer dynamics, *J–V* measurements under various illumination intensities and transient photovoltage (TPV) decays are measured. The LCE-based device achieves a smaller slope of 1.50 *kT*/*q* than that of the reference device (1.92 *kT*/*q*), which indicates that the trap-assisted recombination is effectively suppressed under the open-circuit condition (Supplementary Fig. 19a). The higher α value for the LCE-based device (0.994) means more effective suppression in bimolecular recombination under the short-circuit condition (Supplementary Fig. 19b)^28^. In addition, the photovoltage decay time of the LCE-based device (0.17 ms) is much longer than that of the reference device (0.05 ms) (Supplementary Fig. 20), which suggests less charge recombination in LCE-based device^46^. In summary, the construction of ordered LCE interlayer at the perovskite/ETL interface is beneficial for improving electron extraction and reducing charge accumulation as well as the resultant enhanced device performance.

The operational stability of those PSCs is further investigated by tracking unencapsulated devices at a fixed bias set to the initial maximum power point (MPP) voltage under AM 1.5 G illumination. The reference device rapidly loses 80% of its initial PCE after 19 h of testing under ambient conditions while the LC oligomer-based device remains 76% of the initial PCE after 100 h of testing (Fig. 3d). Notably, the LCE-based device can maintain 92% of its initial PCE after continuous operation for 100 h under AM 1.5 G illumination (Fig. 3d) and additionally show a *T*_80_ lifetime of over 1570 h under flowing N_2_ compared to that of 510 h for the reference device (Fig. 3e). In accordance with the ISOS protocol, we further conduct a continuous MPP tracking to the LCE-based PSC, which retains 85% of the initial performance after aging for 1443 h and shows a *T*_90_ lifetime exceeding 500 h (Supplementary Fig. 21 and Supplementary Table 4). The degradation mechanism is explored by conducting the time evolutional GIWAXS (in Supplementary Fig. 22) and XRD measurements of the reference and LCE-based PSCs under vacuum and 14,740 lux illumination (Supplementary Fig. 23). The two-dimensional GIWAXS pattern (Fig. 3f) and XRD data (Supplementary Fig. 24) of the reference PSC show an evolution at (012) Bragg peak after operation for 90 min, which is attributed to the lattice shrinkage and phase segregation into the minority phase and majority phase during the operation^47^. In contrast, the introduction of the LCE interlayer can effectively cause a compact interfacial contact and suppress the segregation that generally occurs at the SnO_2_/perovskite interface during the operation (Fig. 3g). To further understand the light induced degradation at the bottom interface, focused ion beam (FIB)-SEM and top-view SEM characterizations are carried out. The top-view SEM images are taken by deliberately delaminating one-half of the PSCs. As shown in Fig. 3h, a large amount of small or huge voids are observed in the reference PSC while remarkably, none of such degradation features is seen in the LCE-based PSC, which demonstrates that the LCE interlayer can provide a tight interfacial contact between the perovskite and SnO_2_.

### Performance and integration of flexible PSCs

**Fig. 4:**
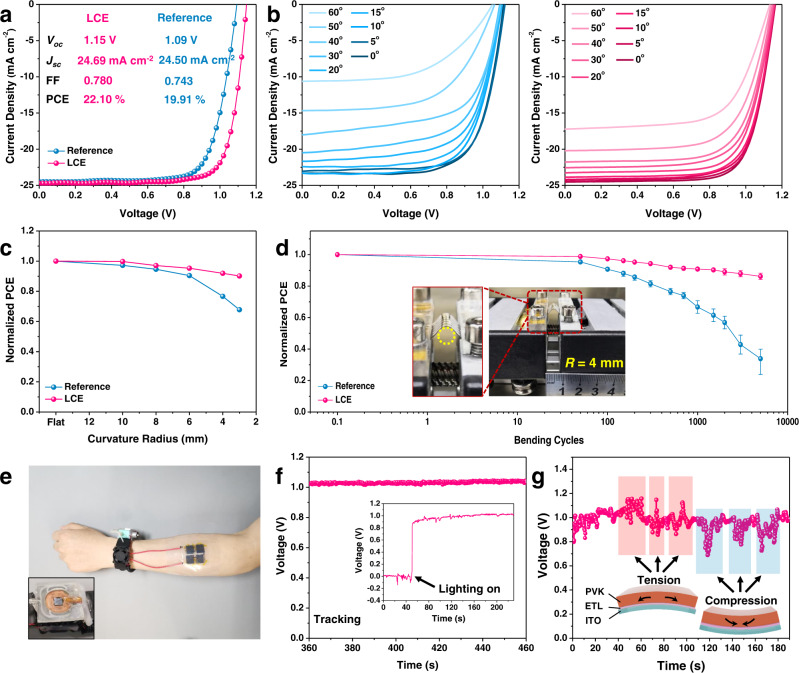
Performance of flexible PSCs. **a** *J–V* curves of the champion flexible PSCs based on LCE and reference, respectively. **b** *J–V* curves measured at different bending angles for reference and LCE-based flexible PSCs. **c** Normalized PCE values of flexible PSCs after bending 500 cycles with different bending radii. **d** Normalized average PCE values for flexible PSCs as a function of bending cycles with the bending radius of 4 mm. **e** Photographic image of the IoT system based on flexible PSCs under ambient light. The inset shows the wearable haptic device. **f** Time-dependent voltage for continuous tracking of the IoT system. The inset shows the light-on photovoltage. **g** Mechanical bending test for the integrated wearable device under continuous illumination. The inset shows the schematic illustrations of the integrated device under tension or compression strain.

Flexible devices with the structure of PEN/ITO/SnO_2_/LCE/CsFAMAPbI_3_/Spiro-OMeTAD/Ag are fabricated to expand the application of the LCE interlayer, which showcases significantly enhanced photovoltaic performance with respect to that of the reference device. As shown in Fig. 4a, the flexible device based on LCE achieves a champion PCE of 22.10% with a *V*_oc_ of 1.15 V, a *J*_sc_ of 24.69 mA cm^−2^, and an FF of 0.780, which is among the highest efficiencies reported for flexible PSCs (Supplementary Table 5). Additionally, the efficiency loss from the rigid substrate to flexible substrate can be minimized to 5%, which is the lowest value reported so far (Supplementary Fig. 25). It is worth noting that the corresponding *J*_sc_ value (24.27 mA cm^−2^) integrated by the EQE spectrum is well-matched with the *J*_sc_ obtained from the *J–V* curve (Supplementary Fig. 26). We further measure the efficiency of the flexible PSCs under the various bending angles from 60° to flat (0°) to investigate the recovery ability after deformation (Fig. 4b, Supplementary Table 6 and Supplementary Table 7). These PCE values for the flexible devices under different bending angles are revised to exclude the impact of effective area variations caused by bending. The reference device loses 7% of its initial PCE when recovering from the bending angle of 60° to 0°, which is typically attributed to the perovskite/ETL interface delaminaion^19,48^. Notably, the flexible device with LCE only loses 4% of the initial PCE after deformation, which demonstrates that the LCE interlayer can enhance the structural integrity of the perovskite/ETL interface thus resulting in PSCs with boosted operational stability. Meanwhile, the relationship between the normalized PCE and various curvature radii (from flat to 3 mm) for flexible PSCs after 500 bending cycles (Fig. 4c) indicates the LCE-based flexible PSC presents better mechanical endurance compared with that of the reference device. The average PCE of the LCE-based flexible device can maintain 86% of its initial PCE value while, however, the average PCE of the reference device shows a significant fall to 34% of its original value after 5000 bending cycles with a bending radius of 4 mm (Fig. 4d). Additionally, cross-sectional SEM images of the LCE-based flexible PSC under the bending condition proves that LCE is beneficial to protect the structural integrity of flexible PSCs (Supplementary Fig. 27). The residual stress caused by the coefficient of thermal expansion (CTE) mismatch with the substrate is also considered an important factor affecting the stability of flexible PSCs. They are typically equi-biaxial in nature and will be superimposed due to the bending stress^8^. Therefore, we use depth-dependent grazing incident X-ray diffraction with the 2*θ*-sin^2^*φ* method^49^ to explore the in-plane residual stress gradient distribution for the perovskite films based on the reference and LCE substrates (Supplementary Fig. 28). Through fitting the functional relationship between 2*θ* and sin^2^*φ*, the slope of the function fitting curves can reflect and calculate the specific value of residual stress^50^. As shown in Supplementary Fig. 29 and Supplementary Note 2, the residual stress result of the perovskite film based on LCE is calculated to be 12.01 MPa, which is significantly smaller than 25.52 MPa for the perovskite film based on the reference SnO_2_. The decreased residual tensile stress suggests reduced lattice distortion and a more stable crystal structure in perovskite film based on the aligned LCE. Hence, the presence of LCE can effectively release the interfacial residual stress at the perovskite/ETL interface by aligned molecular stacking^31^ and reduce the whole stress distribution of ITO and PVK films by constructing elastic interfaces^7^.

To explore the application of the LCE-based flexible PSC in the Internet of Things (IoT) ecosystem^51,52^, the flexible PSC is further integrated with microneedle-based arrays of sensors as a pain sensation system in wearable haptic devices^53^. The whole system is assembled into a watch-like device and triggered in virtual reality scenes (Fig. 4e)^54^ with the LCE-based flexible PSC to power the temperature control system. The continuous photovoltage tracking is recorded under ambient light to assess the reliability of the integrated device and the stabilized photovoltage is observed (~1.03 V) (Fig. 4f and Supplementary Fig. 30) as well. Four temperatures including 20 °C, 25 °C, 30 °C, and 35 °C are modulated to induce different pain levels at given capsaicin concentrations. The integrated device exhibits excellent stability for monitoring temperatures constantly at four levels (Supplementary Fig. 31) and variably during the heating process from 30 °C to 35 °C (Supplementary Fig. 32), respectively. Moreover, the mechanical bending test for the integrated wearable device under continuous illumination is performed to simulate the real-life operation (Fig. 4g). The output photovoltage changes under both tension and compression strain, which is mainly caused by the variation of effective illumination area under ambient light, and will recovers once the deformation is removed. To better understand the mechanism of the performance recovery, the morphology changes regarding the interface during bending cycles are further investigated (Supplementary Fig. 33). Regarding 2500 bending cycles, the perovskite film begins to peel off from the SnO_2_ layer (Supplementary Fig. 33a), while the SnO_2_/LCE sample almost shows no morphological changes (Supplementary Fig. 33b). After bending for 5000 cycles, the reference SnO_2_/perovskite film exhibits an obvious structural failure (Supplementary Fig. 33c) while the SnO_2_/LCE/perovskite film only shows slight cracks and interfacial fatigue (Supplementary Fig. 33d). Fatigue-resistant adhesion interfaces can be constructed by anchoring ordered nanostructures, which requires much higher energy for fatigue-crack propagation than amorphous surface structures. Thus, tough adhesion between SnO_2_ and perovskite materials can be achieved by anchoring elastic polymer chains of aligned LCE on SnO_2_ thin-film surfaces (Supplementary Fig. 33e). Such tough adhesion suffers from fatigue dissipation over multiple cycles of mechanical loads, which is commonly occurred in adhesive interfaces^55^. In essence, these fatigue dissipations come from the interfacial tensile stress and will not significantly hinder the longitudinal transport of photogenerated carriers. To further investigate the structural integrity, the overhead cross-sectional SEM measurement is conducted (Supplementary Fig. 34). Compared to the reference SnO_2_/perovskite film, the SnO_2_/LCE/perovskite film shows a complete structure, which manifests that the device based on LCE can maintain better performance. The satisfactory PCE and excellent stability of LCE-based perovskite devices confirm the significant role of LCE in enhancing perovskite solar cell reliability and benefiting its practical applications.

## Discussion

We have demonstrated the LCE interlayer of PSCs formed by instant locking of molecular ordering, which remarkably facilitates the rapid charge extraction and the minimal charge recombination at the interface between SnO_2_ and CsFAMA perovskite films. The LCE interlayer makes it possible to manufacture PSCs with a maximum PCE of 23.26% and 22.10% on the rigid and flexible substrates, respectively. The suppression of phase segregation due to the presence of the LCE interlayer enables unencapsulated PSCs to show a *T*_80_ lifetime of over 1570 h under flowing N_2_. Moreover, the enhanced structural integrity endows the flexible devices with excellent repeatability, stability, and mechanical endurance (86% of the initial efficiency after 5000 bending cycles). A wearable haptic device is demonstrated by integrating the LCE-based PSC with microneedle-based arrays of sensors. The finding provides an effective strategy for the design of reliable interfaces for perovskite photovoltaic and flexible electronics technologies.

## Methods

### Materials

Formamidinium iodide (FAI) (≥99.5% purity), methylamine iodide (MAI) (≥99.5% purity), cesium iodide (CsI) (≥99.5% purity), lead (II) iodide (PbI_2_, >99.999% purity), 2,2’,7,7’tetrakis-[N,N-di(4-methoxyphenyl)-amino]-9,9’-spirobifluorene (Spiro-OMeTAD), bis(trifluoromethane) sulfonimide lithium salt (Li-TFSI) and 4-*tert*butylpyridine (*t*BP) were purchased from Xi’an p-OLED Corp. The SnO_2_ colloid precursor was obtained from Alfa Aesar. 1,8-diazabicycloundec-7-ene (DBU), 1,3-pentanedithiol, 2-methyl-1,4-phenylene bis(4-(3-(acryloyloxy)propoxy)benzoate) (RM257) were purchased from Aladdin Reagents. 2,2-dimethoxy-2-phenylacetophenone (DMPA, 99%), (3-Aminopropyl)triethoxysilane) (APTES ≥ 98%), chlorobenzene (anhydrous, 99.8%), acetonitrile (anhydrous, 99.8%), *N*,*N*-dimethylformamide (DMF, anhydrous 99.8%) and dimethyl sulfoxide (DMSO, ≥99.5%) were purchased from Sigma-Aldrich.

### Synthesis and preparation of LCE

Synthesis of LC oligomer. LC oligomers were synthesized as previously published^36^. LC diacrylate (RM257) and 1,3-pentanedithiol were added into CH_2_Cl_2_ at a molar ratio of 1:2 with stirring. Two drops of DBU were then added as the catalyst. The reaction mixture was stirred at room temperature overnight. The mixture was washed with diluted HCl aqueous solution twice (first 1 M then 0.1 M) followed by washing with deionized water once. The CH_2_Cl_2_ solution was then dried by anhydrous MgSO_4_ powder for 30 min and filtrated to remove MgSO_4_. The final LC oligomer was collected as a viscous liquid after evaporating the CH_2_Cl_2_ solvent under vacuum.

Synthesis and preparation of LCE. RM257 and LC oligomers were mixed at 1:1 molar ratio in toluene followed by addition of 2 wt% DMPA as the photoinitiator and 0.2 wt% BHT as the inhibitor. The concentration of LCE was controlled within 0.05-0.5 mg mL^−1^ by adding different amounts of prepolymer in toluene. The mixture was spin-coated on the SnO_2_-coated substrates at 6000 rpm for 30 s. Then, the as-deposited films were moved to a 150 °C hot plate and annealed for 30 min to remove the solvent and achieve uniform LC texture. Finally, the LCE films were obtained by exposing the mixture-coated substrate to UV lamp for 10 min to complete the polymerization.

### Device fabrication

The indium tin oxide (ITO)-coated glass substrates or PEN/ITO (Peccell, Japan) substrates were cleaned with acetone, detergent water, deionized water and isopropanol and dried with a nitrogen (N_2_) stream. For rigid solar cells, the SnO_2_ nanoparticles solution (2.67%) was spin-coated on UV-ozone-treated ITO/glass substrates at 5000 rpm for 30 s, followed by thermal annealing at 150 °C for 30 min. For flexible solar cells, glass/polydimethylsiloxane (PDMS) was used as the support. The PEN/ITO was attached on the PDMS during the whole fabrication process to guarantee uniform deposition. The SnO_2_-coated PEN/ITO was annealed at 120 °C for 30 min. The ordered LCE layer was prepared as mentioned above. The CsFAMA perovskite precursor solution was prepared by mixing 1.25 M PbI_2_, 0.87 M FAI, 0.25 M MAI, and 0.13 M CsI in DMF/DMSO (4:1, v/v) with stirring at room temperature for 2 h. To optimize the perovskite crystalline quality on flexible substrates, 0.02 wt% polyurethane (PU) was added into the perovskite precursor^25^. The perovskite layer was obtained by spin-coating the precursor solution at 1000 rpm for 10 s and then 5000 rpm for 30 s. At the 32^nd^ second of spinning, 200 μL of anti-solvent chlorobenzene was dripped at the center. Subsequently, the as-deposited films were annealed at 100 °C for 1 min and 120 °C for 30 min. The Spiro-OMeTAD solution was prepared by dissolving 72.3 mg Spiro-OMeTAD in 1 mL chlorobenzene. 17.5 μL Li-TFSI solution (520 mg LiTFSI in acetonitrile) and 28.8 μL of *t*BP were added to the solution. The Spiro-OMeTAD layer was prepared by spin-coating the solution at 3000 rpm for 30 s. Finally, 90 nm Ag layer was thermally evaporated on the top of Spiro-OMeTAD layer as an electrode.

### Materials characterizations

Scanning electron microscopy (SEM) images were obtained by field emission scanning electron microscope (Hitachi, SU8020) at an acceleration voltage of 5 kV. The FIB-SEM images were obtained using FEI Helios Nanolab G3 CX System. A 0.2-μm-thick Pt layer was deposited to prevent damage from Ga-ion imaging or milling. The exposure of bottom perovskite for X-ray photoelectron spectroscopy (XPS) analysis is achieved by ion beam miller machine (Leica EM RES101). Sections of samples with areas of 1 cm^2^ were milled using an accelerating voltage of 1 kV in a working pressure of 10^−5^ mbar for 2 min. A slow rotation was used to ensure a uniform milled surface with an argon gas flow during etching. XPS analysis was performed on the Thermo Scientific ESCALab 250Xi using 200 W monochromatic Al Kα radiation. The 500 μm X-ray spot was used for SAXPS analysis. The base pressure in the analysis chamber was about 3 × 10^−9^ mbar. Typically, the hydrocarbon C1s line at 284.8 eV from adventitious carbon was used for energy referencing. Fourier-transform infrared (FTIR) spectra were recorded in transmittance mode using IR spectrometer instrument (Bruker, Tensor-27). Atomic force microscopy (AFM) images were obtained using MultiMode 8-HR (Bruker). X-ray diffraction (XRD) measurements were recorded by D8-Discover 25 diffractometer (Bruker). Grazing incidence wide-angle X-ray scattering (GIWAXS) measurements were performed by a Xeuss 2.0 spectrometer (Xenocs Company) with MetalJet-D2 (Excillum) as the X-ray source and Pilatus 3R 1M (Dectris) as the detector. GIXRD measurements were recorded on D8-Discover 25 diffractometer (Bruker) and characterization with 2*θ*-sin^2^*φ* method at an incident angle of 0.3°. The ultraviolet-visible (UV-Vis) spectra were recorded by SHIMADZU, UV-2600 spectrophotometer. Steady-state photoluminescence (PL) and time-resolved photoluminescence (TRPL) spectra were recorded by a fluorescence spectrometer (FLS980, Edinburgh Instruments Ltd.).

### Solar cells characterizations

The current density–voltage (*J*–*V*) characteristics were measured by the Keithley 2400 source meter under simulated AM 1.5 sunlight at 100 mW cm^−2^ irradiance generated using an Enli Tech solar simulator and the reference silicon solar cell was corrected from the National Renewable Energy Laboratories (NREL) to accurately estimate the equivalent AM 1.5 irradiance level. Before the measurement of each effective device, the intensity of the solar simulator was automatically detected by using the above reference silicon solar cells to calculate the precise power conversion efficiency (PCE). The forward *J–V* scans were measured from forward bias to short circuit and the backward scans were from short circuit to forward bias, both with a delay time of 30 ms, a scan rate of 0.2 V s^−1^, and 8.0 mV for each step. The area of PSCs was corrected by calibrated apertures (0.16 cm^2^). The external quantum efficiency (EQE) spectra were recorded with QE-R systems (Enli Tech. Co. Ltd.). The SCLC measurement was recorded by the Keithley 4200-SCS under air condition and the trap density of states was deduced from the angular frequency-dependent capacitance. All the measurements were performed under a nitrogen atmosphere at room temperature.

### Stability measurement

The in situ GIWAXS studies on the CsFAMA PSCs were done under continuous illumination. For continuous operational stability test, the *J–V* curves were recorded first to verify the voltage at the maximum power point (MPP). Then the operational stability tests were carried out at the MPP for unencapsulated devices under 1-sun intensity continuous white LED illumination. Note that during the whole MPP tracking, the illumination was continuously applied on the PSCs except for the calibration of light source. Reverse scanned *J–V* curves are measured every three hours.

### Reporting summary

Further information on research design is available in the Nature Portfolio Reporting Summary linked to this article.

## Supplementary information

Supplementary Information

Solar Cells Reporting Summary

## Data Availability

The data that support the findings of this study are available from the corresponding authors upon request. Source data of Figs. 1f, 2a, 2d–g, 3a–e, 4a–d, 4f, 4g, Supplementary Figs. 1, 4, 5, 9, 11–21,24, 26, 29–32, Supplementary Tables 1–7 is provided in the Source data file. Source data are provided with this paper.

## Source data

Source Data
