# Supplementary material for: Wearable perovskite solar cells by aligned liquid crystal elastomers
Source: Nat Commun. 2023 Mar 2;14:1204. doi: 10.1038/s41467-023-36938-7 (PMC9981560; doi:10.1038/s41467-023-36938-7)
Supplement: Supplementary file 1 — Supplementary Information [file 41467_2023_36938_MOESM1_ESM.pdf]

Supplementary information

Wearable perovskite solar cells by aligned liquid crystal elastomers

Zengqi Huang[1,2], Lin Li[*3], Tingqing Wu[1,4], Tangyue Xue[5], Wei Sun[6], Qi Pan[1,4], Huadong Wang[1,4], Hongfei Xie[1,4], Jimei Chi[1,4], Teng Han[6], Xiaotian Hu[7], Meng Su[*1,4], Yiwang Chen[*2], Yanlin Song[*1,4]

[1] Key Laboratory of Green Printing, Institute of Chemistry, Chinese Academy of Sciences (ICCAS), Beijing Engineering Research Center of Nanomaterials for Green Printing Technology, Beijing National Laboratory of Molecular Sciences (BNLMS), Beijing 100190, P. R. China.
[2] Key Laboratory of Fluorine and Silicon for Energy Materials and Chemistry of Ministry of Education, College of Chemistry and Chemical Engineering, Jiangxi Normal University, 99 Ziyang Avenue, Nanchang, 330022, P. R. China.
[3] Research Center for Green Printing Nanophotonic Materials, School of Materials Science and Engineering, Suzhou University of Science and Technology, Suzhou, 215009, P. R. China.
[4] University of Chinese Academy of Sciences, Beijing 100149, P. R. China.
[5] School of Materials Science and Engineering, Zhengzhou University, Zhengzhou 450001, P.R. China.
[6] Institute of Software, Chinese Academy of Sciences (ISCAS), Beijing 100190, P.R. China.
[7] College of Chemistry, Nanchang University, 999 Xuefu Avenue, Nanchang 330031, P.R. China.

[*]E-mail: ylsong@iccas.ac.cn (Y.S.); ywchen@ncu.edu.cn (Y.C.); sumeng1988@iccas.ac.cn (M.S.); linli@usts.edu.cn (L.L.)

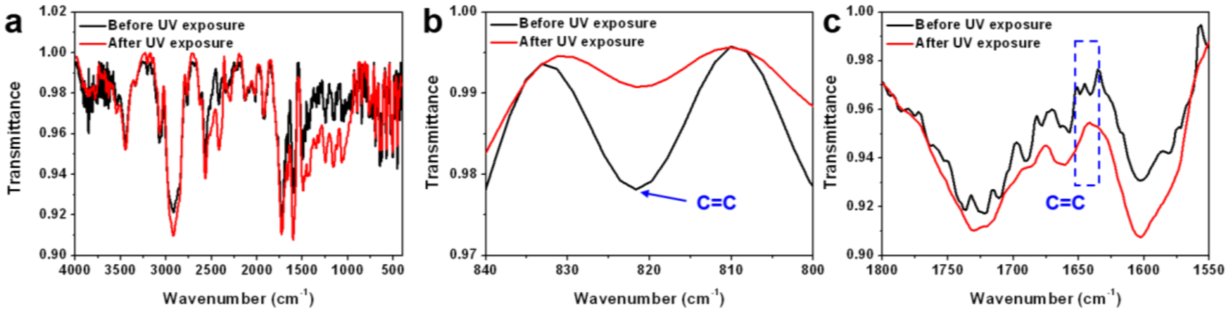

Supplementary Fig. 1. FTIR spectra of the thiol-acrylate reaction by UV exposure. a Full FTIR spectra of the RM257-1,3DT/RM257 system. **b, c** Conversion of the reactants estimated from the change of the FTIR absorption peaks at ~821 cm^{-1} and 1641 cm^{-1} for C=C stretching. The change of FTIR spectra regarding C=C stretching before and after UV exposure indicates the formation of LCE via polymerization of acrylate monomers.

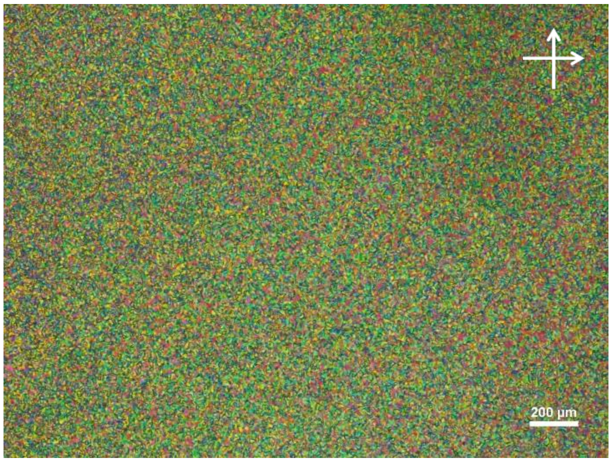

Supplementary Fig. 2. POM image of LCE sandwiched in a cell observed under crossed polarizers. Scale bar: 200 μm. The appearance of the birefringence texture indicates the nematic liquid crystal phase.

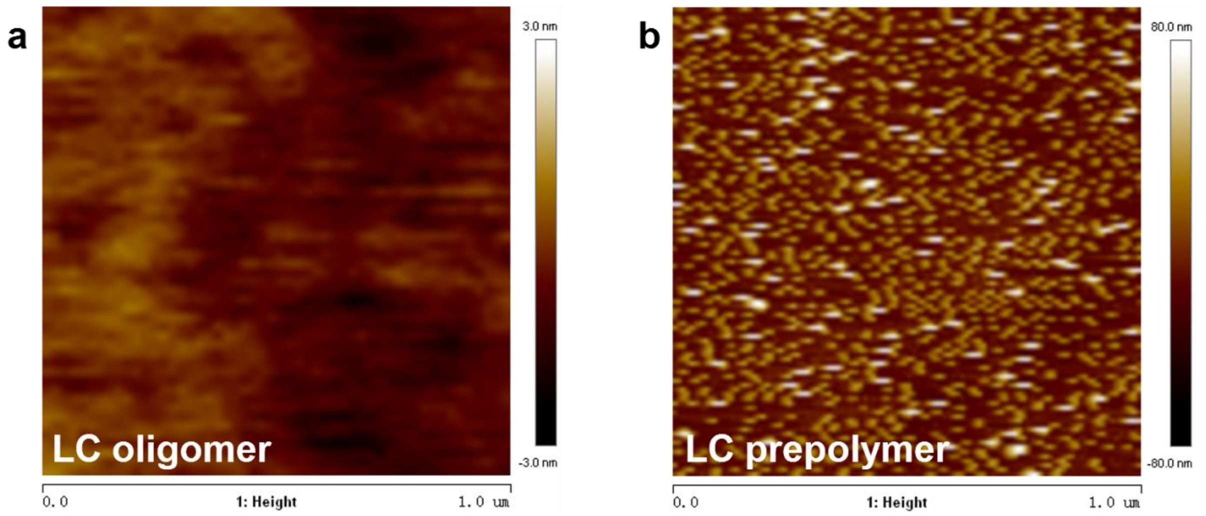

Supplementary Fig. 3. Morphologies of the LC films. AFM height images of **a** LC oligomer and **b** LC prepolymer on ITO glass.

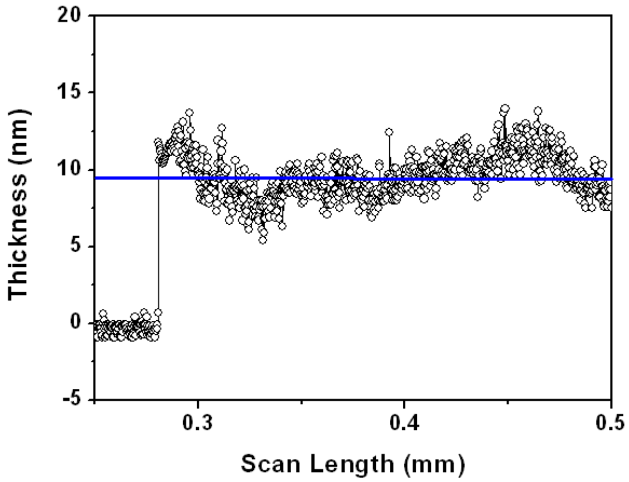

Supplementary Fig. 4. Thickness of the aligned LCE layer deposited on glass/ITO. The mean value of the film thickness is 9.6 nm. The data fluctuation mainly results from the edge scribing and the rough substrate.

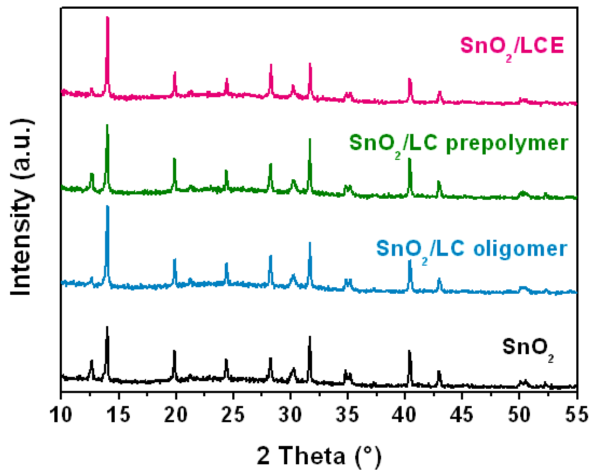

Supplementary Fig. 5. XRD patterns of the perovskite films fabricated on SnO_2, SnO_2/LC oligomer, SnO_2/LC prepolymer, and SnO_2/LCE. The diffractograms of all the samples show the same trigonal perovskite crystal structure. The full-width-at-half-maximum (FWHM) of the (001) perovskite lattice reducing from 0.167 for the perovskite sample on SnO_2 to 0.148 for the perovskite on SnO_2/LCE proves the enhanced crystallinity of the perovskite film. The reduced diffraction peak intensity of PbI_2 indicates that the suppressed formation of PbI_2 in the perovskite surface. In addition, the XRD peak intensity ratio of (001) versus (012) is boosted for the perovskite film fabricated on SnO_2/LCE, which demonstrates the preferential orientation of perovskite crystal growth on the LCE interlayer.

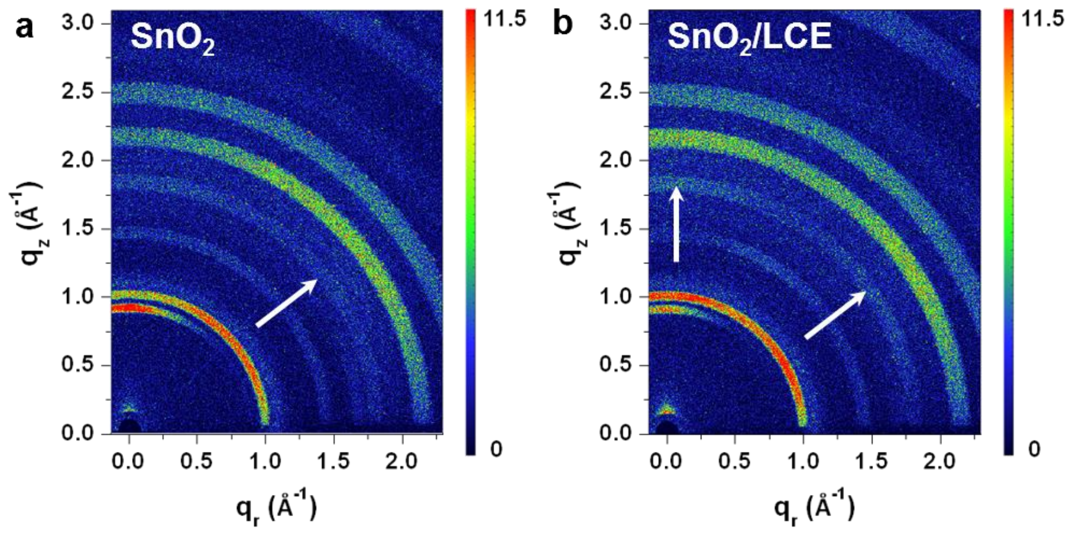

Supplementary Fig. 6. 2D GIWAXS patterns of perovskite films. 2D GIWAXS patterns of the perovskite films fabricated on **a** SnO_2 and **b** SnO_2/LCE. Compared to the perovskite film fabricated on SnO_2, the perovskite film fabricated on SnO_2/LCE exhibits a significant out-of-plane signal along the z axis, which is consistent with the preferential orientation of perovskite crystal growth in XRD measurements.

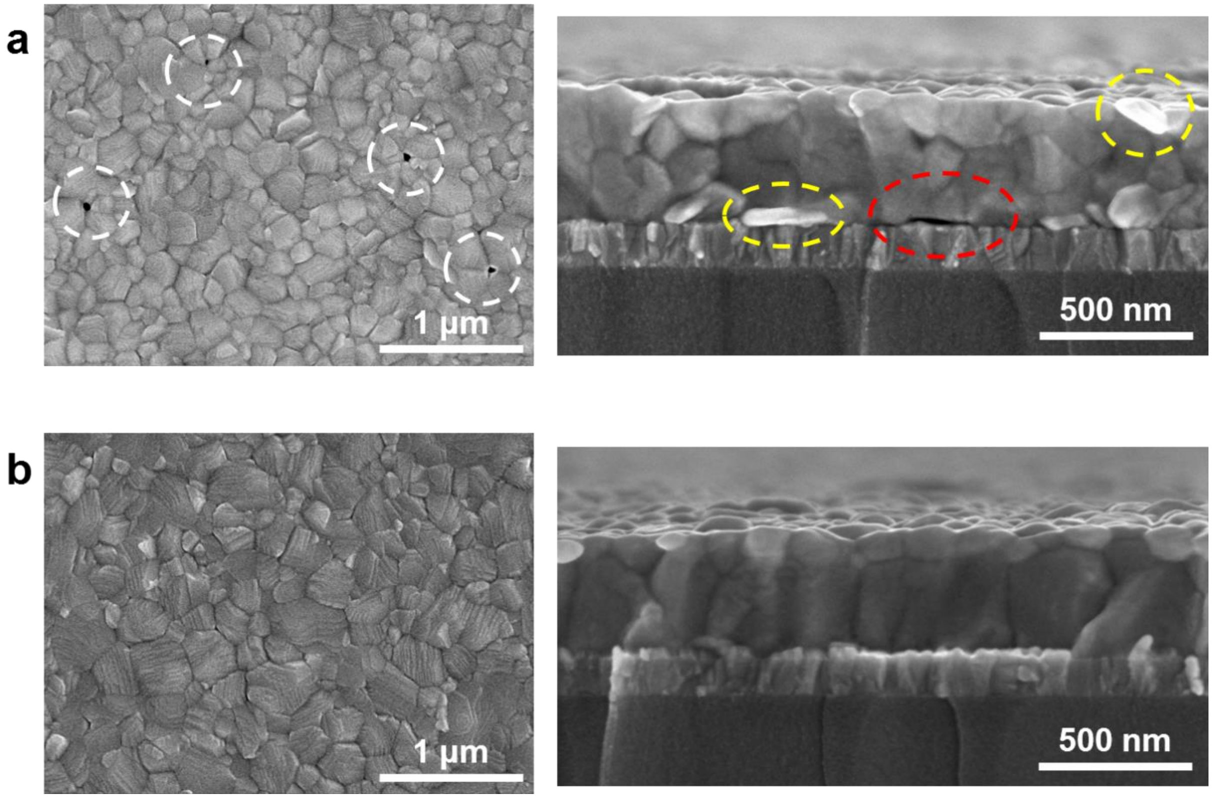

Supplementary Fig. 7. Top-view and cross-sectional SEM images of perovskite films. SEM images of perovskite films fabricated on **a** SnO_2 and **b** SnO_2/LCE interlayers. The white, red, and yellow dashed circles represent pin holes, interface voids, and PbI_2 crystals, respectively.

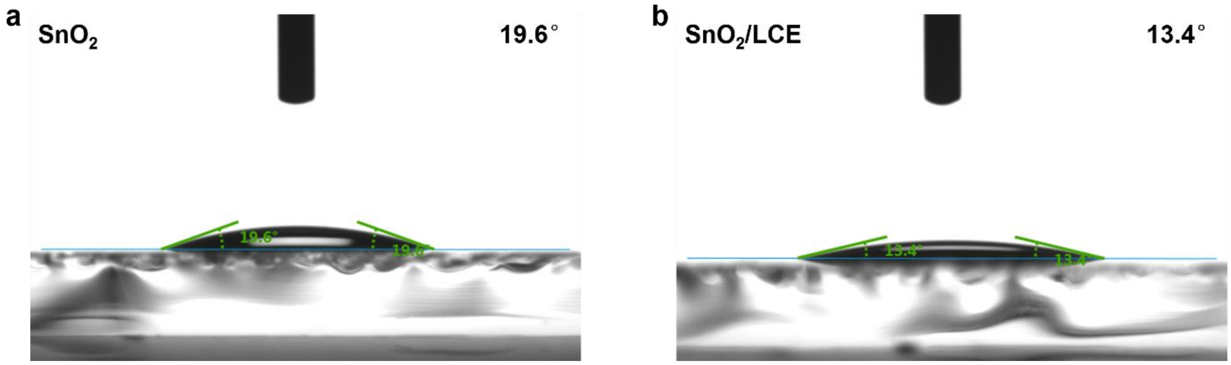

Supplementary Fig. 8. Contact angle analysis. The water contact angles of **a** SnO_2 and **b** SnO_2/LCE. The smaller contact angle can result in a smaller $f(\theta)$ and reduced Gibbs free energy, thereby assisting the nucleation process for the heterogeneous nucleation.

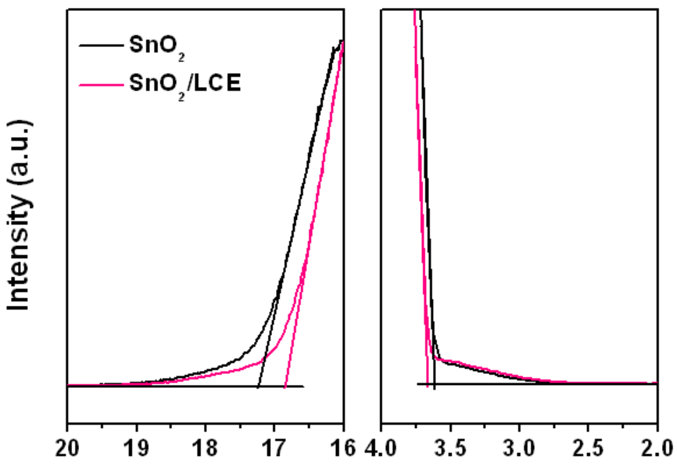

Supplementary Fig. 9. UPS spectra of the SnO_2 and SnO_2/LCE films: the cutoff region (left) and Fermi edge region (right). Compared with the pristine SnO_2, the relative downshift of the CBM in the SnO_2/LCE film facilitates more efficient electron injection from the perovskite into the ETL.

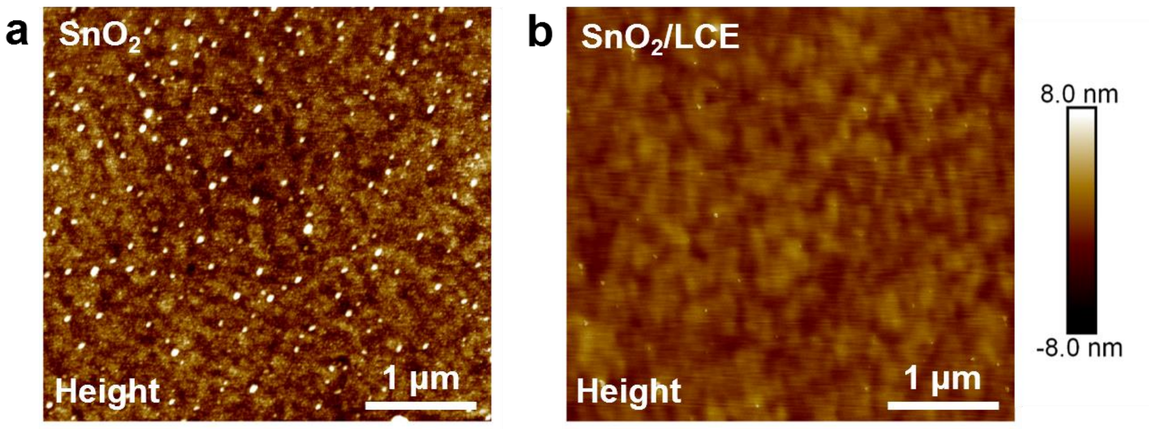

Supplementary Fig. 10. Morphologies of the SnO_2 films. AFM height images of the **a** SnO_2 and **b** SnO_2/LCE films, respectively.

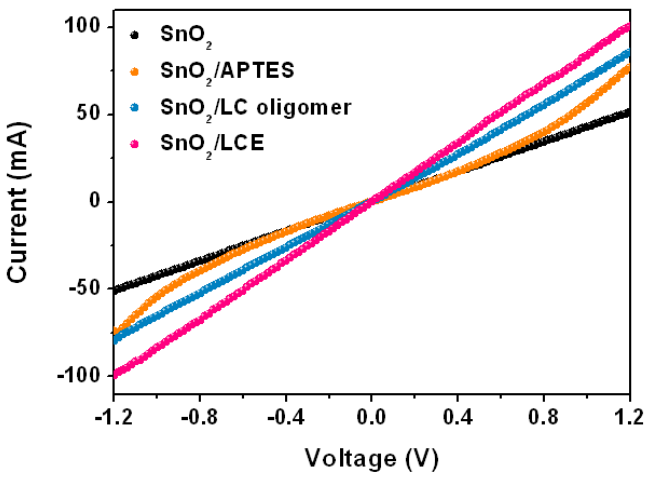

Supplementary Fig. 11. *I-V* curves of devices based on SnO_2, SnO_2/APTES, SnO_2/LC oligomer, and SnO_2/LCE, respectively. The device based on SnO_2/LCE exhibits more efficient charge transfer.

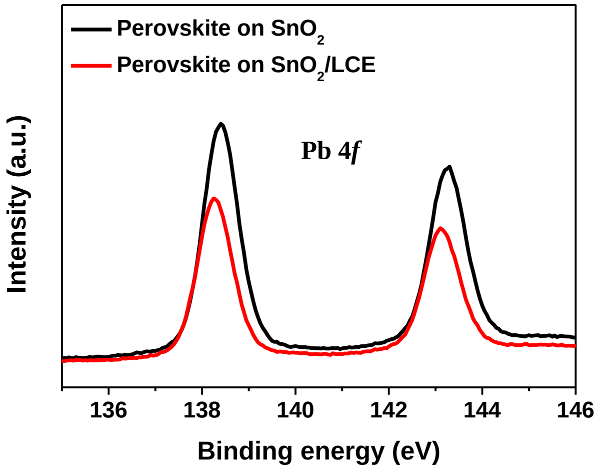

Supplementary Fig. 12. XPS spectra of the Pb signals in the perovskite films fabricated on SnO_2 and SnO_2/LCE, respectively. Compared to the perovskite film on SnO_2, the binding energy for the SnO_2/LCE based perovskite film with respect to Pb $4f_{7/2}$ and Pb $4f_{5/2}$ is observed to be decreased by 0.2 eV, indicating the oxidation state of lead is lowered due to the electron donation from sulfur atom.

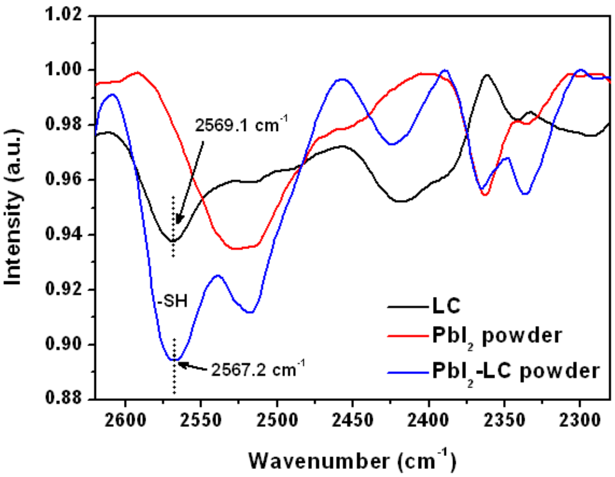

Supplementary Fig. 13. FTIR spectra of LC, PbI_2 powder, and PbI_2-LC powder, respectively. The stretching vibration of –SH in LC observed at 2569.1 cm^{-1} shifts to a lower wavenumber of 2567.2 cm^{-1} in PbI_2-LC powder, which indicates a strong interaction of –SH with PbI_2 by sharing the lone pair electron on the S atom in –SH with the empty 6p orbital of Pb^{2+}.

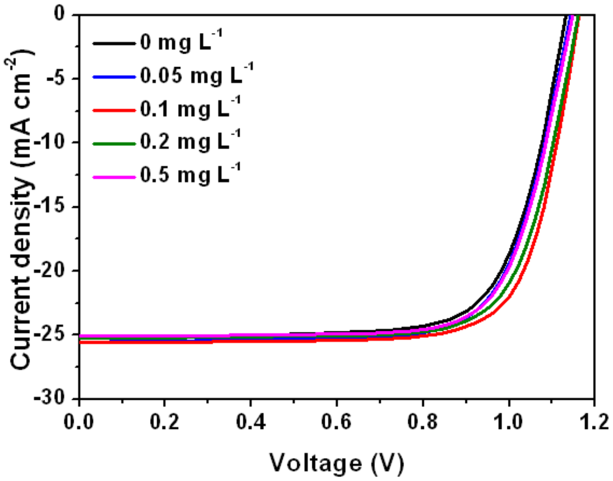

Supplementary Fig. 14. *J*-*V* curves of PSCs based on different LCE concentrations. The optimized LCE concentration for fabricating PSCs is 0.1 mg mL^{-1}. This concentration is utilized for all the device's performance characterizations.

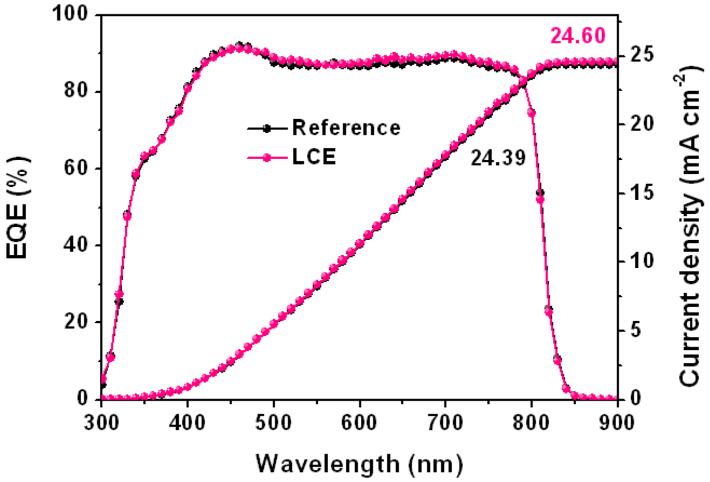

Supplementary Fig. 15. EQE spectra and integrated J_{sc} of PSCs based on reference and LCE. The integrated J_{sc} values of PSCs based on reference and LCE are 24.39 mA cm^{-2} and 24.60 mA cm^{-2}, respectively.

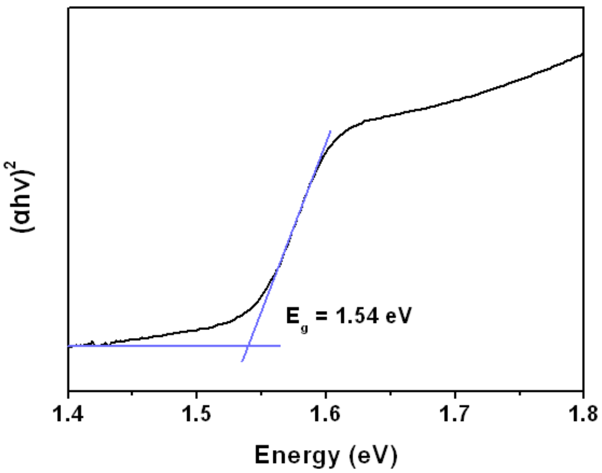

Supplementary Fig. 16. The optical bandgap of the CsFAMA perovskite film. The optical bandgap is determined to be 1.54 eV.

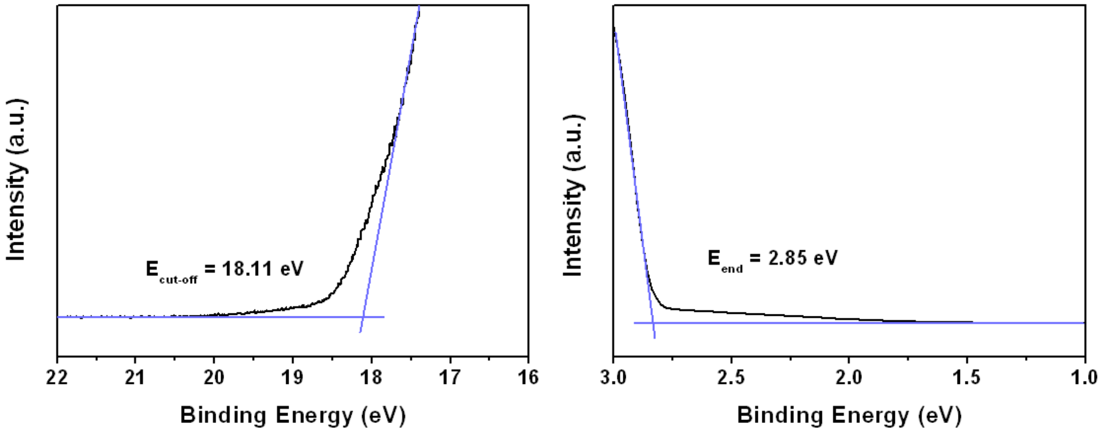

Supplementary Fig. 17. UPS spectra of the CsFAMA perovskite film. The CBM of the perovskite film is determined to be -4.42 eV.

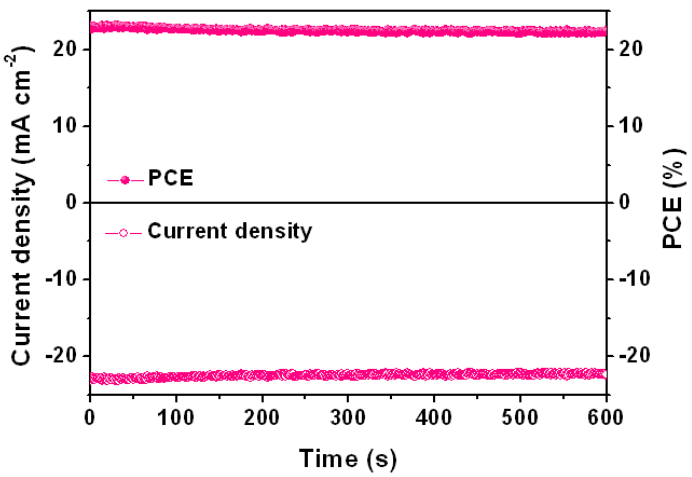

Supplementary Fig. 18. Steady-state photocurrent and output power at the maximum power point for LCE based PSCs. The LCE based device delivers a stabilized PCE of 22.38% at the maximum power point.

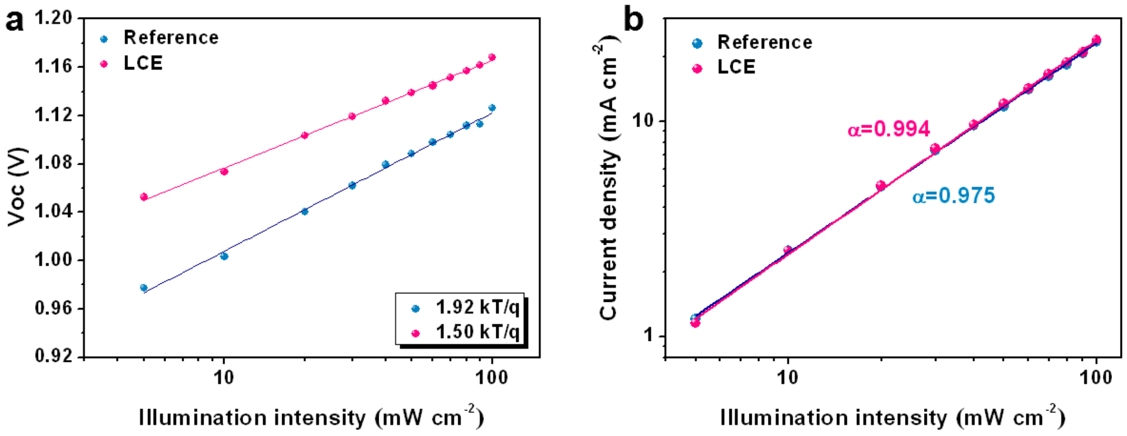

Supplementary Fig. 19. V_{oc} and J_{sc} dependence of PSCs based on reference and LCE. a The slope of V_{oc} versus illumination intensity produced kT/q, where k is the Boltzmann constant, T is the temperature in Kelvin and q is the elementary charge. The slope of LCE based device (1.50 kT/q, pink) is smaller than that of the reference device (1.92 kT/q, blue), which indicates that the trap-assisted recombination is effectively suppressed under the open-circuit condition. **b** The power law dependence of J_{sc} on the illumination intensity can be denoted as $J_{sc} \propto I^{\alpha}$, where I and α are the light intensity and exponential factor, respectively. The α value for the LCE based device (0.994) being higher than that of the reference device (0.975) demonstrated a more effective suppression in bimolecular recombination under the short-circuit condition.

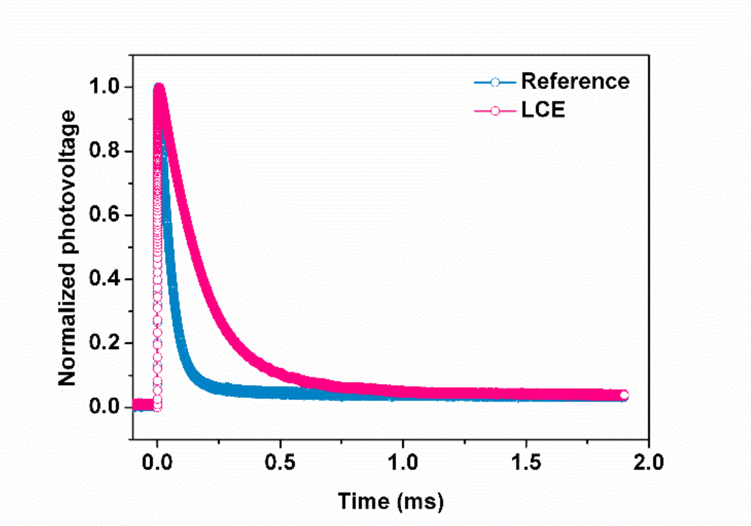

Supplementary Fig. 20. TPV results of PSCs based on reference and LCE. The photovoltage signals are triggered by a pulse light that pumps up the carrier density and thus quasi-Fermi level splitting and charge recombination are directly measured via monitoring the photovoltage decay[1,2]. The experimental results are fitted by a bi-exponential decay function. The photovoltage decay time of the LCE based device (0.17 ms) is much longer than that of the reference device (0.05 ms). The prolonged photovoltage decay time suggests less charge recombination in the LCE based device, both at the SnO_2/LCE/perovskite interface and in the bulk perovskite, which is mainly attributed to efficient passivation of the trap states on the surface of the SnO_2 ETL, as well as the improved quality of corresponding perovskite film.

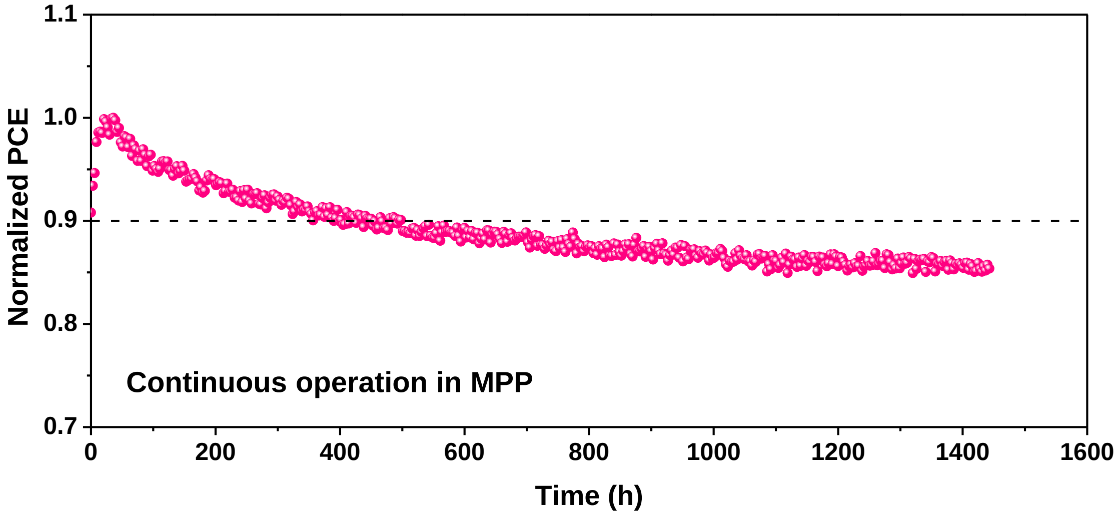

Supplementary Fig. 21. Continuous MPP tracking measured for the LCE based PSCs. The MPP measurement is performed under the following conditions: 1-sun continuous illumination, unencapsulated, flowing N_2 atmosphere, at room temperature. The LCE based PSCs can retain 85% of the initial performance after aging for 1443 h and shows a T_{90} lifetime exceeding 500 h.

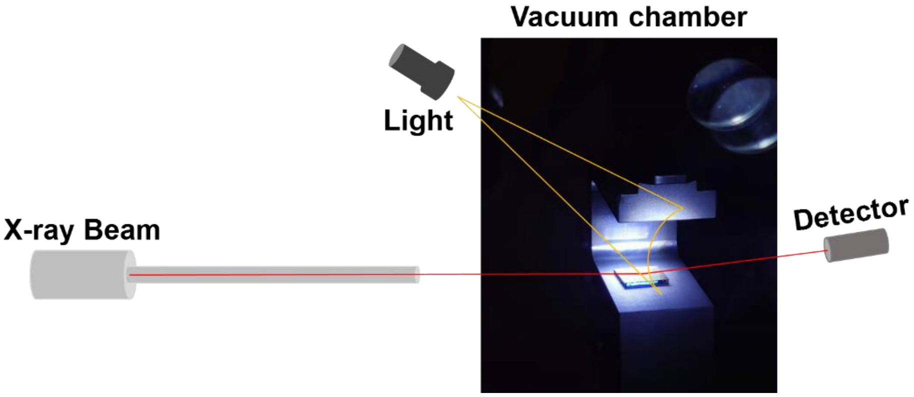

Supplementary Fig. 22. Schematic illustration of the time evolutional GIWAXS measurement under continuous illumination. A continuous illumination is loading on the perovskite sample in the vacuum chamber. The X-ray source and detector are located on the left and right sides of the vacuum chamber, respectively.

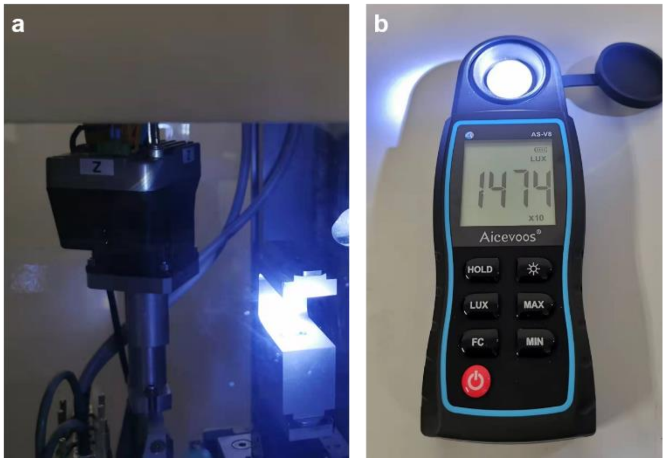

Supplementary Fig. 23. The illumination setup images. Photographic images of **a** the illumination setup under vacuum and **b** the corresponding fixed intensity (~14,740 lux).

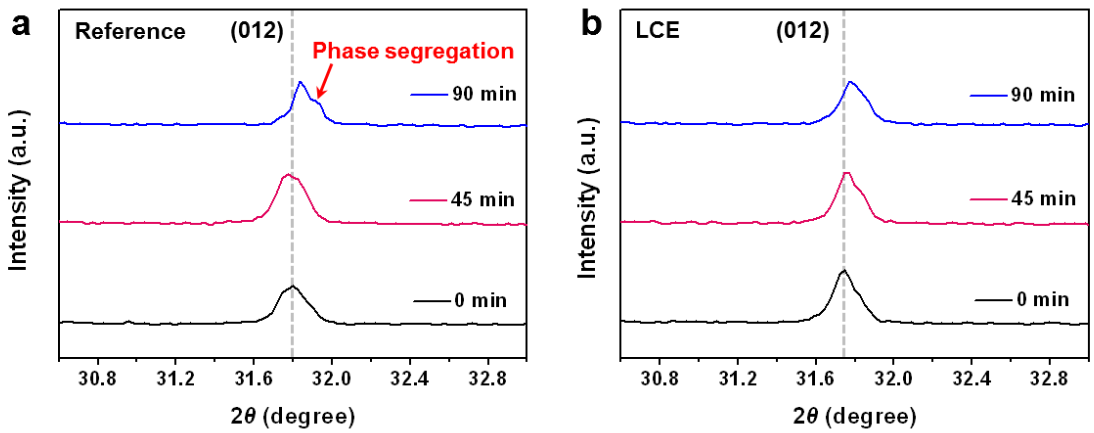

Supplementary Fig. 24. XRD patterns of perovskite films. Time evolutional XRD patterns of perovskite films based on **a** reference and **b** LCE. The diffraction peak (012) of CsFAMA perovskite splits up with the increase of the operation time under vacuum, indicating a phase segregation and the formation of the new component $FAPbI_3$ after 90 min operation under vacuum. The introduction of the LCE interlayer can effectively cause a compact interfacial contact and suppress this segregation that generally occurs at the SnO_2/perovskite interface during the operation.

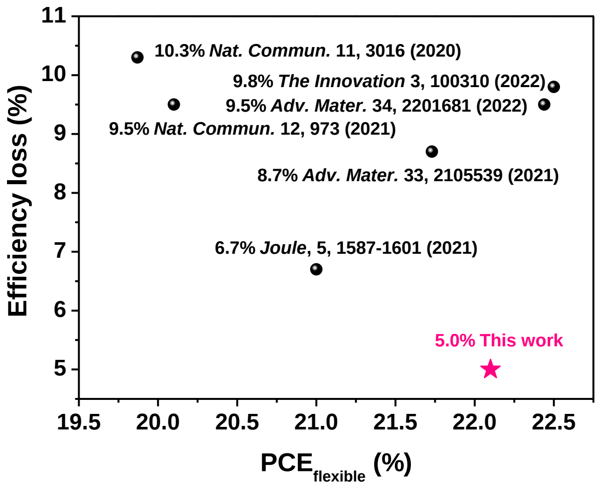

Supplementary Fig. 25. The efficiency loss in recently reported PSCs from the rigid substrates to the flexible substrates for comparison to this work. The efficiency loss can be determined by the following equation: $\text{Efficiency loss} = \frac{\text{PCE}_{\text{rigid}} - \text{PCE}_{\text{flexible}}}{\text{PCE}_{\text{rigid}}}$.

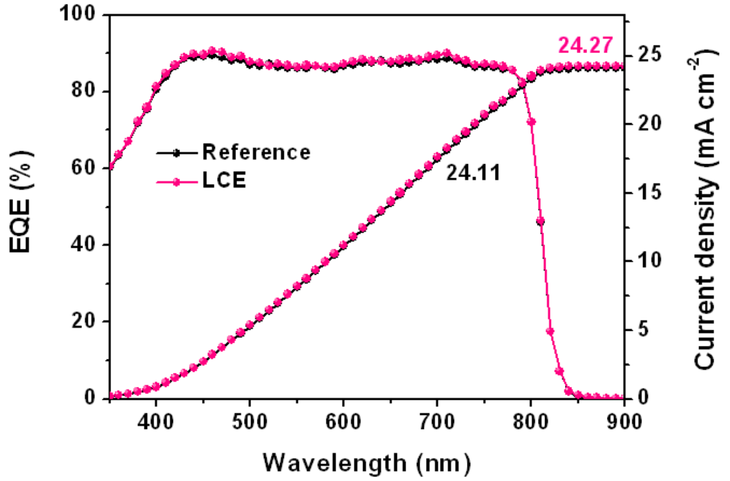

Supplementary Fig. 26. EQE spectra and integrated J_{sc} of flexible PSCs based on reference and LCE. The integrated J_{sc} values of flexible PSCs based on reference and LCE are 24.11 mA cm^{-2} and 24.27 mA cm^{-2}, respectively.

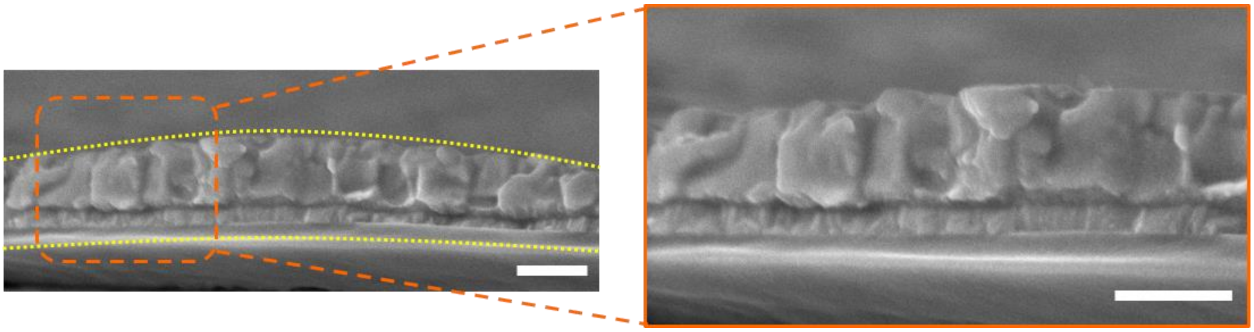

Supplementary Fig. 27. Cross-sectional SEM images of the LCE based flexible PSC under the bending condition. Scale bar: 500 nm. The structural integrity of flexible PSCs can be protected by the introduction of LCE.

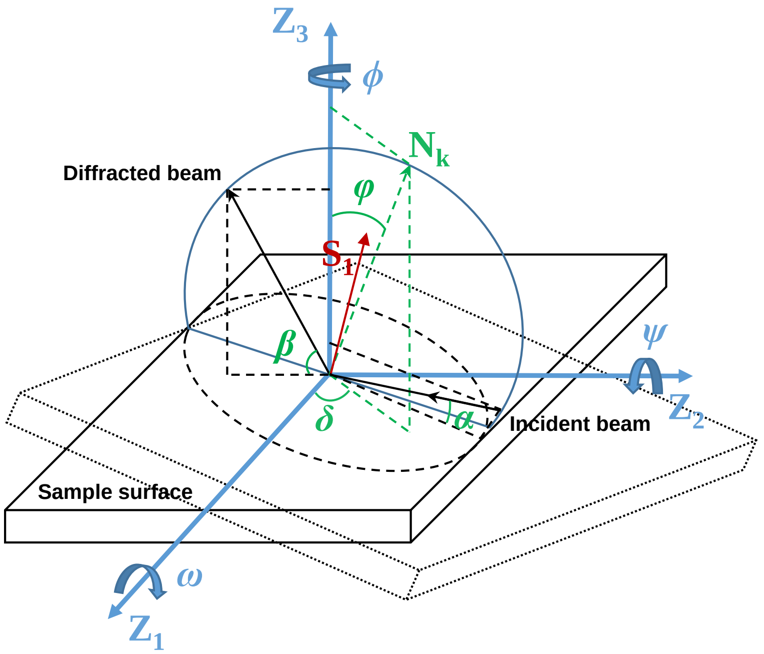

Supplementary Fig. 28. The diffraction geometries of the depth-dependent strain distribution measurement. The relation between the instrument reference (Z) frame and the diffraction vector (α and β are the incident and exit angles of X-rays, defined as the angle between the incident/diffracted X-rays and the sample surface).

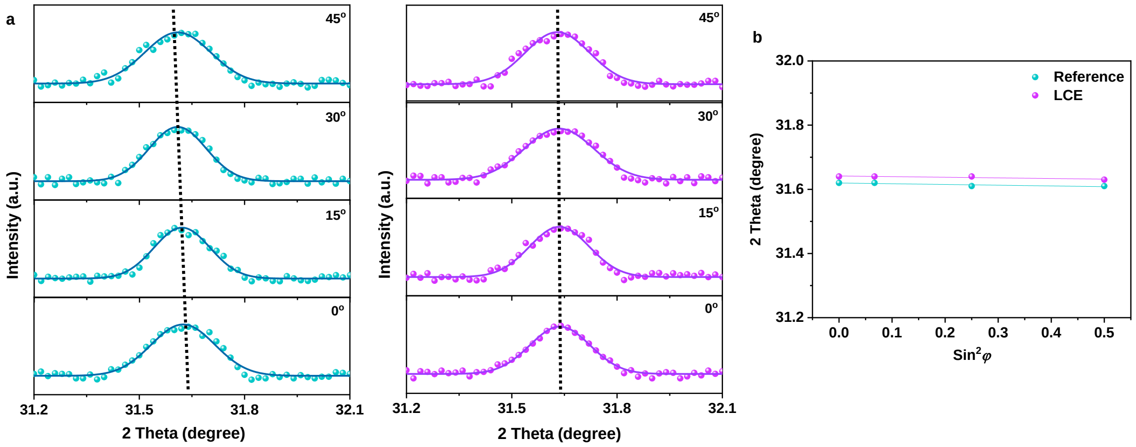

Supplementary Fig. 29. **Residual strain distribution measurement with the GIXRD method.** **a** The GIXRD patterns at different tilt angles for the perovskite films based on the reference and LCE substrates. **b** Residual strain distribution for the corresponding perovskite films. The measured (points) and Gauss fitted (line) diffraction strain data as a function of $\sin^2\varphi$.

The film stress can be calculated by fitting 2θ as a function of $\sin^2\varphi$[3,4], in which the negative slope of the fitting line indicates tensile stress that exists at the perovskite film formed on PEN/ITO/ETL flexible substrate. As shown in Supplementary Fig. 29, the residual stress result of the perovskite film based on LCE is calculated to be 12.01 MPa, which is significantly smaller than 25.52 MPa for the perovskite film based on the reference SnO_2. The decreased residual tensile stress suggests the reduced lattice distortion and a more stable crystal structure in perovskite film based on the aligned LCE, which is potentially beneficial for enhancing efficiency and stability in corresponding flexible PSCs[5,6]. The supplementary test information is detailed in Supplementary Note 2.

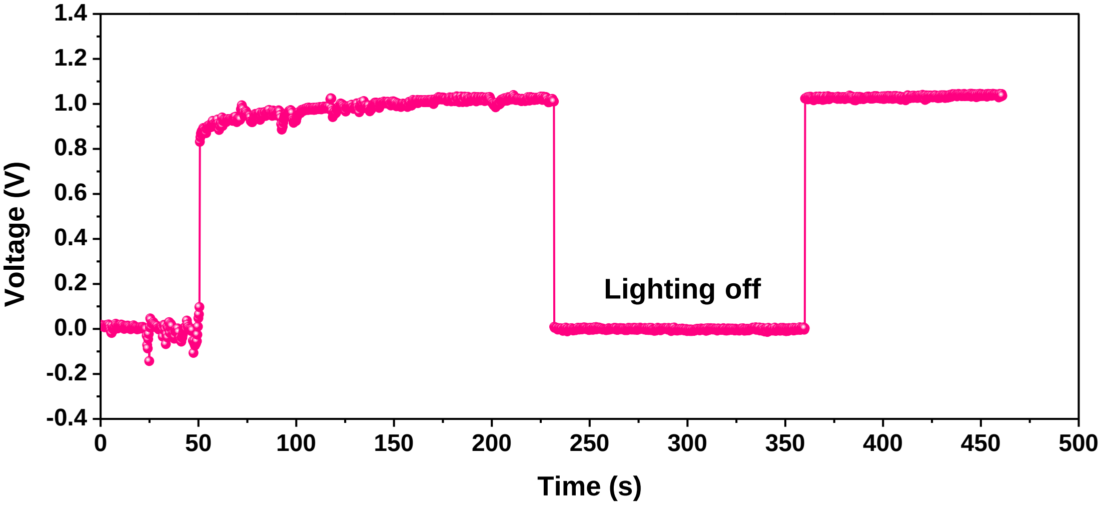

Supplementary Fig. 30. **Time-dependent voltage for continuous tracking of the IoT system under light on-off switching.** The continuous photovoltage tracking is recorded under ambient light to assess the reliability of the integrated device.

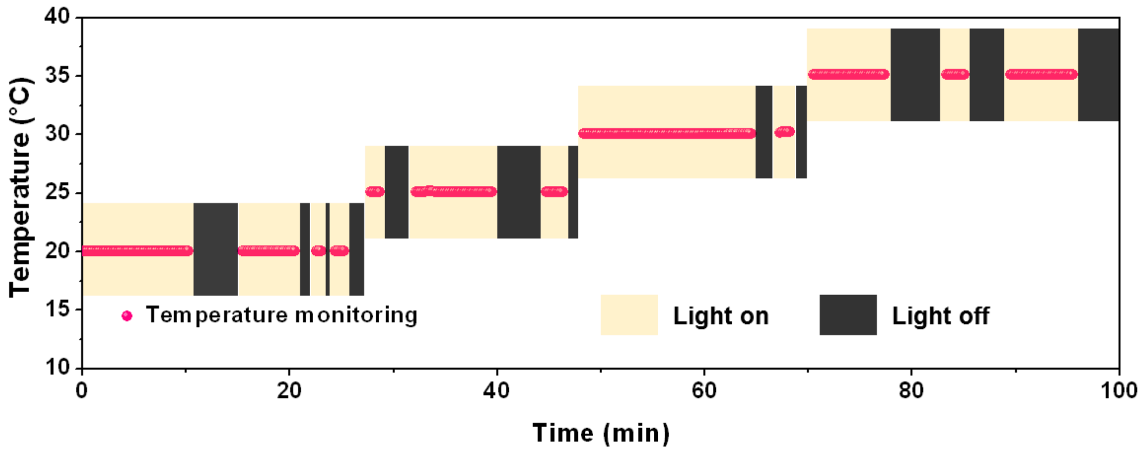

Supplementary Fig. 31. Light on-off switching test for temperature monitoring at each level. The integrated device exhibits excellent stability for monitoring temperatures constantly at four temperatures including 20 ℃, 25 ℃, 30 ℃, and 35 ℃.

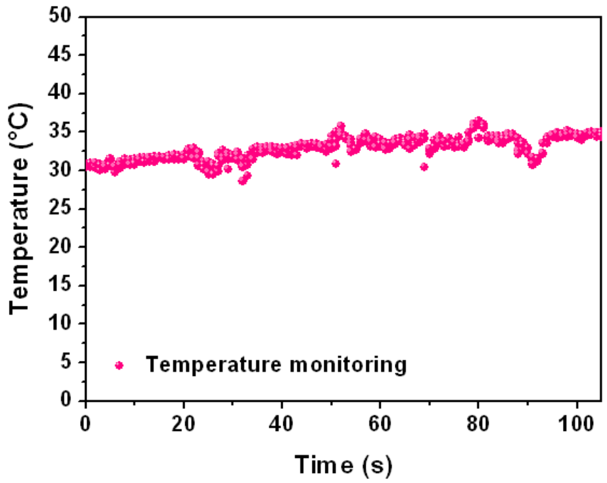

Supplementary Fig. 32. Temperature monitoring for the continuous heating process. The temperature changes can be fully monitored by the integrated devices.

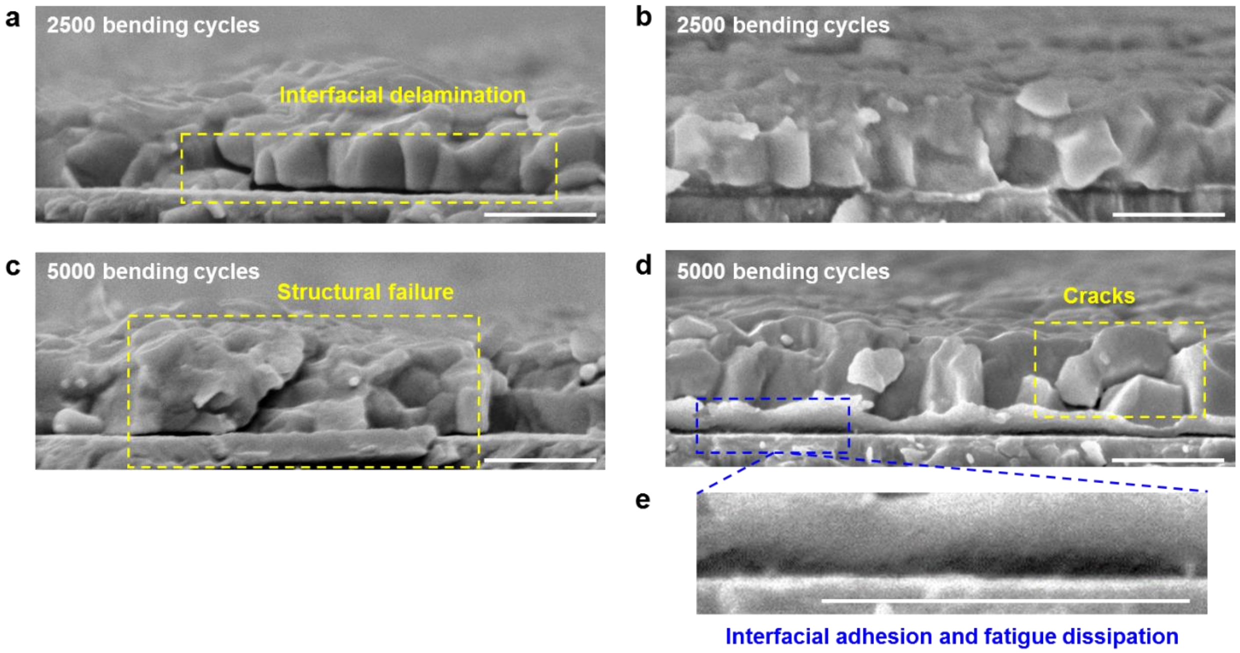

Supplementary Fig. 33. Morphology changes regarding the interface during bending cycles. Cross-section SEM images of **a**, **c** PEN/ITO/SnO_2/perovskite film and **b, d** PEN/ITO/SnO_2/LCE/perovskite film measured after bending cycles of 2500 and 5000, respectively. **e** The interfacial adhesion and fatigue dissipation between SnO_2/LCE and perovskite layer. Scale bar: 500 nm. The perovskite film begins to peel off from the SnO_2 layer (Supplementary Fig. 33a) while the SnO_2/LCE sample almost shows no morphological changes under 2500 bending cycles (Supplementary Fig. 33b). After bending for 5000 cycles, the reference SnO_2/perovskite film exhibits an obvious structural failure (Supplementary Fig. 33c) while the SnO_2/LCE/perovskite film only shows slight cracks and interfacial fatigue (Supplementary Fig. 33d). Fatigue-resistant adhesion interfaces can be constructed by anchoring ordered nanostructures, which requires much higher energy for fatigue-crack propagation than amorphous surface structures. Thus, tough adhesion between SnO_2 and perovskite materials can be achieved by anchoring elastic polymer chains of aligned LCE on SnO_2 thin-film surfaces (Supplementary Fig. 33e). Such tough adhesion suffers from fatigue dissipation over multiple cycles of mechanical loads, which is commonly occurred in adhesive interfaces. These fatigue dissipations come from the interfacial tensile stress and will not significantly hinder the longitudinal transport of photogenerated carriers.

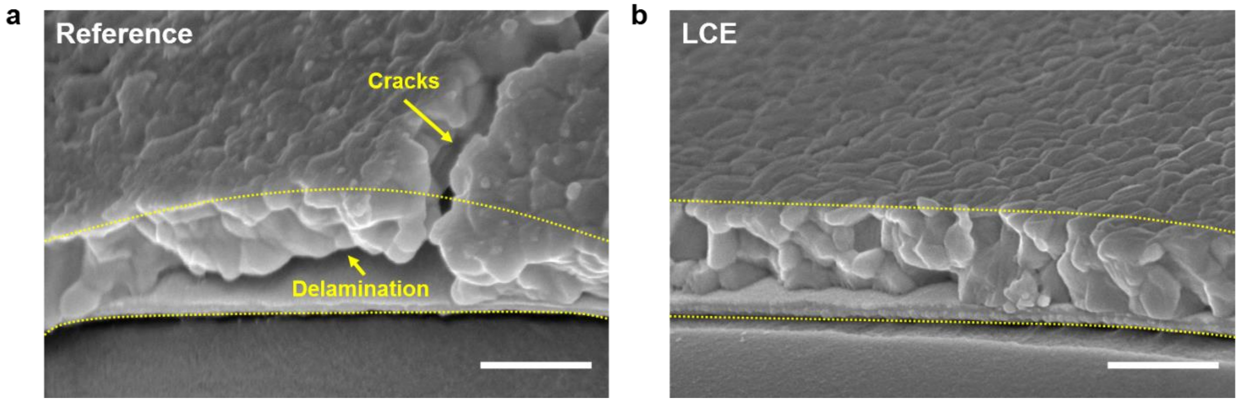

Supplementary Fig. 34. Structural integrity of perovskite films. Overhead cross-sectional SEM images of **a** PEN/ITO/SnO_2/perovskite film and **b** PEN/ITO/SnO_2/LCE/perovskite film (after 5000 bending cycles) measured under the bending condition, respectively. Scale bar: 500 nm. Compared to the reference SnO_2/perovskite film, the SnO_2/LCE/perovskite film shows a complete structure.

Supplementary Table 1. The characteristic peak intensities and FWHM in the XRD patterns.

**Samples**	**FWHM**		**Intensity**		**Intensity ratio**
	**(001)**	**(012)**	**(001)**	**(012)**	
SnO_2	0.167	0.161	550	469	1.17
SnO_2/LC oligomer	0.174	0.163	772	465	1.66
SnO_2/LC prepolymer	0.176	0.142	663	538	1.23
SnO_2/LCE	0.148	0.153	773	380	2.03

Supplementary Table 2. Fitted parameters for the time-resolved photoluminescence (TRPL) decay measurements for perovskite films on various substrates.

Substrate	τ_1 (ns)	A_1 (%)	τ_2 (ns)	A_2 (%)	τ_{ave} (ns)[a]
ITO Glass	28.5	38.9	1018.7	61.1	633.5
SnO_2	2.8	50.2	36.1	46.5	18.8
SnO_2/LC oligomer	2.2	64.6	33.8	31.2	12.5
SnO_2/Mixture	3.0	64.3	48.8	35.3	19.2
SnO_2/LCE	2.4	40.0	18.9	57.6	12.1

[a] Average decay time was calculated based on the equation: $\tau_{ave} = (A_1\tau_1 + A_2\tau_2)/(A_1 + A_2)$

Supplementary Table 3. Photovoltaic parameters determined from the *J-V* curve in Fig. 3a.

Device	J_{sc} (mA cm^{-2})	V_{oc} (V)	FF	PCE (%)
Reference	23.86±0.85	1.10±0.02	0.69±0.07	19.40±1.04
	24.67	1.13	0.74	20.62
SnO_2/LC oligomer	24.22±0.55	1.11±0.02	0.70±0.04	20.14±0.99
	24.72	1.14	0.75	21.18
SnO_2/LCE	24.50±0.36	1.16±0.01	0.78±0.02	22.47±0.38
	24.79	1.17	0.80	23.26

The average and standard deviation values were based on measures of 50 cells. The sign "±" was defined as the error bar.

Supplementary Table 4. The performance of recently reported unencapsulated PSCs (n-i-p structure) for comparison to this work.

Structure	Remaining PCE of the initial	Operation time	Reference
FTO/FI-SnO_2/ Perovksite/Spiro-OMeTAD/Au	~82%	1000 h	*Nat. Commun.* **12**, 973 (2021)
ITO/SnO_2-EDTAK/Perovskite (EAMA)/PCBM/BCP/Ag	~80%	~1600 h	*Nat. Energy* **5**, 596-604 (2020)
ITO/SnO_2/I-SAM/ Perovskite/Spiro-OMeTAD/Au	~88%	~1300 h	*Science,* **372**, 618-622 (2021)
ITO/Cl-bSO/ Perovskite/Spiro-OMeTAD/Au	~90%	500 h	*Nature* **598**, 444-450 (2021)
ITO/SnO_2/Perovskite/ Spiro-OMeTAD/Au	~80%	852 h	*Nat. Commun.* **13**, 4891 (2022)
ITO/paa-QD-SnO_2@c-TiO_2/Perovskite/OAI/ Spiro-OMeTAD/Au	~84%	350 h	*Science* **375**, 302-306 (2022)
**ITO/SnO_2/LCE/$CsFAMAPbI_3$/ Spiro-OMeTAD/Ag**	**~90%**<br>**~85%**	**500 h**<br>**1443 h**	**This work**

Supplementary Table 5. The performance of recently reported flexible devices for comparison to this work.

Structure	PCE (%)	Remaining PCE of the initial	Bending radius (mm)	Bending cycles	Ref.
**PEN/ITO/SnO_2/LCE/$CsFAMAPbI_3$/Spiro-OMeTAD/Ag**	**22.10**	**86%**	**4**	**5000**	**This work**
PEN/ITO/SnO_2/Perovskite/Spiro-OMeTAD/Ag	19.51	94.9%	8	6000	7
PEN/ITO/PEDOT:PSS/PTAA/$MAPbI_3$/PC_{61}BM/BCP/Ag	19.05	90%	3	2000	8
PET/PEDOT:PSS:CFE/PEDOT:PSS/Perovskite/PCBM/Ag[a]	19.0	85%	3	5000	9
PEN/ITO/FI-SnO_2/Perovksite/Spiro-OMeTAD/Au	20.1	85%	-	2500	10
MgF_2/Willow Glass/ITO/PTAA/$MAPbI_3$/C_{60}/BCP/Cu	19.72	-	-	-	11
PET/ITO/PEDOT:EVA/Perovskite/PCBM/BCP/Ag[a]	19.87	85%	3	7000	12
PEN/ITO/HfO_x/SnO_2/ Perovskite/Spiro-OMeTAD/Au	21.10	93.2%	10	2000	13
PEN/ITO/PTAA/Perovskite/Organic BHJ/Zr(acac)$_4$/Ag	21.73	≈95%	-	1000	14
PET/ITO/HADI-SnO_2/$FA_{0.9}Cs_{0.1}PbI_3$/Spiro-OMeTAD/Au[b]	22.44	93%	5	1000[b]	15

[a] Our previous report on flexible PSCs based on p-i-n structures. [b] Diagonal bending cycles.

Supplementary Table 6. Photovoltaic parameters of flexible PSCs based on reference measured at different bending angles.

Bending Angles	J_{sc} (mA cm^{-2})	V_{oc} (V)	FF	PCE (%)
60 °	10.63	1.05	0.55	6.12
50 °	14.67	1.07	0.59	9.27
40 °	18.01	1.10	0.63	12.52
30 °	20.49	1.09	0.64	14.37
20 °	21.68	1.10	0.66	15.77
15 °	22.40	1.10	0.68	16.95
10 °	23.31	1.10	0.69	17.82
5 °	23.05	1.12	0.71	18.41
0 °	23.32	1.11	0.71	18.56
Pristine	24.50	1.09	0.74	19.91

Supplementary Table 7. Photovoltaic parameters of flexible PSCs based on LCE measured at different bending angles.

Bending Angles	J_{sc} (mA cm^{-2})	V_{oc} (V)	FF	PCE (%)
60 °	17.23	1.13	0.65	12.70
50 °	20.21	1.14	0.67	15.50
40 °	21.78	1.15	0.70	17.37
30 °	22.56	1.15	0.70	18.24
20 °	23.27	1.16	0.71	19.22
15 °	23.87	1.16	0.73	20.07
10 °	24.19	1.16	0.73	20.48
5 °	24.43	1.16	0.73	20.86
0 °	24.53	1.16	0.74	21.13
Pristine	24.69	1.15	0.78	22.10

Supplementary Note 1.

Calculations of the trap density. At intermediate voltages, the trap-filled limit (TFL) regime was identified by a rapid nonlinear rise in the current where trap states were filled by the injected carriers. And the onset voltage (V_{TFL}) is proportional to the density of traps (n_t). The concentrations of trap states can be determined by equation (1).

$$V_{TFL} = \frac{en_tL^2}{2\varepsilon\varepsilon_0} \tag{1}$$

where e represents the elementary charge, L represents the film thickness, ε is the relative dielectric constant of perovskite, and ε_0 is the vacuum permittivity. The V_{TFL} of the SnO_2/LCE based device (0.17 V) is much lower than that of the SnO_2 based device (0.41V), leading to a decreased trap density by an order of magnitude from 1.16×10^{16} cm^{-3} to 6.68×10^{15} cm^{-3} (Fig. 2f).

In addition, thermal admittance spectroscopy measurement has been carried out to evaluate the trap density of states (*t*DOS). The *x*-axis is converted from frequency to energy by equations (2) and (3). The differentiated capacitance spectra at a specific temperature are superimposed to yield the energetic defect distribution (Equation (4)).

$$E\omega = k_B T \ln\frac{\omega_0}{\omega} \tag{2}$$

$$\omega = 2\pi f \tag{3}$$

$$N_T(E\omega) = -\frac{V_{bi}}{qW}\frac{dC}{d\omega}\frac{\omega}{k_BT} \tag{4}$$

where q is the elementary charge, k_B is the Boltzmann's constant, T is the temperature, ω is the angular frequency, C is the capacitance. W and V_{bi} represent the depletion width and built-in potential, respectively, which are extracted from the Mott-Schottky analysis. Generally, the trap density region between 0.30-0.40 eV is ascribed to the shallow trap states, which mainly exist at GBs. While the deeper trap region above 0.40 eV is assigned to the deep trap states, which mainly come from the film surface[16]. The lower *t*DOS in the SnO_2/LCE based device is related to the improved quality of perovskite film with reduced grain boundary and pin holes (Fig. 2g).

The lower trap density indicates that the photogenerated carriers could shift through the device without encountering defects and recombination, which results in longer recombination lifetimes and higher open-circuit voltage.

Supplementary Note 2.

Note for depth resolved GIXRD measurement. The penetration depth and measuring direction are held constant by varying the three instrumental angles (ω, ψ, ϕ). When the stress is tested using grazing incident method, the incident angle α and the tilt angle φ are no longer equal to ω and ψ. To describe the relationships of the instrumental angles (ω, ψ, ϕ, θ) and diffraction geometry angles ($\alpha, \beta, \varphi, \delta$), the instrument coordinate system（Z_1, Z_2, Z_3）and sample reference system(S_1, S_2, S_3) are introduced in Supplementary Fig. 28. The X-ray penetration depth is determined with the incident and exit angles of the X-rays, which can be replaced by the instrumental angles[17]:

$$t = \cos\psi\,[\sin^2\theta - \sin^2(\theta-\omega)]/[2\mu\sin\theta\ \cos(\theta-\omega)] \tag{5}$$

In this formula (5), the penetration depth is a function of the instrumental angles (ω, ψ, θ). Taking into account about 500 nm thick perovskite film, the information depth τ_t, is determined with the instrumental angles (ω, ψ, θ), linear absorption coefficient μ and the sample thickness t in this study[18]:

$$\tau_t = \frac{1}{k\mu} + \frac{t}{1-\frac{1}{\exp(-\mu k t)}} \tag{6}$$

$$\text{with } k = \frac{2\sin\theta\cos(\theta-\omega)}{\cos\psi[\sin^2\theta - \sin^2(\theta-\omega)]}$$

The relationships between the diffraction geometry angles (φ, δ) and the instrumental angles (ω, ψ, ϕ, θ) are described with the formulas as follows[17]:

$$\cos\varphi = \cos\psi\cos(\omega-\theta) \tag{7}$$

$$\delta = \phi + \arctan[-\sin\psi\ /\ \tan(\omega-\theta)] \tag{8}$$

when determining the crystal face(2θ is determined), a series of instrumental angles ψ are proposed for a defined angular zone, the series of incident angles ω can be calculated from equation (6) for a selected depth τ_t. Thus, the polar angles φ can be obtained from equation (7) with the calculated value of ω. In formula (8), the azimuth angle φ remains constant by varying the instrumental angle ϕ.

Furthermore, we assume that the perovskite film is in a quasi-isotropic stress state. According to the Bragg's Law and Hooke's Law, the classic $\sin^2\varphi$ equation about stress σ and 2θ can be obtained as follow[19,20]:

$$\sigma = -\frac{E}{2(1+\nu)}\frac{\pi}{180}\cot\theta_0\frac{\partial(2\theta)}{\partial\sin^2\varphi} \tag{9}$$

where E and υ are Young's modulus (10 GPa) and Poisson's ratio (0.3) of the perovskite thin films, respectively[3,21,22]. θ_0 is half of the scattering angle $2\theta_0$ corresponding to a given diffraction peak for stress free perovskite, $2\theta_0$, 31.6 °[23]. 2θ is the diffraction peak for the actual perovskite thin films. φ is the angle of the diffraction vector with respect to the sample surface normal. The film stress can be calculated from formula (9) through fitting the 2θ as a function of $\sin^2\varphi$. The slope of the fitted line represents the scale of the residual strain, the negative slope indicates the films bear tension stress[4]. Thus, by adjusting the incident angles from 0.1 °to 1 °, the structural differences in the perovskite layer can be revealed at a theoretical probing depth of ~ 30 nm to 3000 nm[24,25].

Supplementary References

1. Abate, A. et al. Supramolecular halogen bond passivation of organic-inorganic halide perovskite solar cells. *Nano Lett*. **14**, 3247-3254 (2014).
2. Liu, K. et al. Fullerene derivative anchored SnO_2 for high-performance perovskite solar cells. *Energy Environ. Sci.*, **11**, 3463-3471 (2018).
3. Zhang, Y. et al. Molecularly tailored SnO_2/perovskite interface enabling efficient and stable $FAPbI_3$ solar cells. *ACS Energy Lett.* **7**, 929-938 (2022).
4. Zhu, C. et al. Strain engineering in perovskite solar cells and its impacts on carrier dynamics. *Nat. Commun.* **10**, 815 (2019).
5. Liu, D. et al. Strain analysis and engineering in halide perovskite photovoltaics. *Nat. Mater.* **20**, 1337 (2021).
6. Zheng Z. et al. Pre-buried additive for cross-layer modification in flexible perovskite solar cells with efficiency exceeding 22%. *Adv. Mater.* **34**, 2109879 (2022).
7. Huang, K. et al. High-performance flexible perovskite solar cells via precise control of electron transport layer. *Adv. Energy Mater.* **9**, 1901419 (2019).
8. Wang, Z. et al. Rational interface design and morphology control for blade-coating efficient flexible perovskite solar cells with a record fill factor of 81%. *Adv Funct. Mater.* **30**, 2001240 (2020).
9. Hu, X. et al. A mechanically robust conducting polymer network electrode for efficient flexible perovskite solar cells. *Joule* **3**, 2205-2218 (2019).
10. Dong. Q. et al. Interpenetrating interfaces for efficient perovskite solar cells with high operational stability and mechanical robustness. *Nat. Commun.* **12**, 973 (2021).
11. Dai, X. et al. Scalable fabrication of efficient perovskite solar modules on flexible glass substrates. *Adv. Energy Mater.* **10**, 1903108 (2020).
12. Meng, X. et al. Bio-inspired vertebral design for scalable and flexible perovskite solar cells. *Nat. Commun.* **11**, 3016 (2020).
13. Yang, L. et al. Artemisinin-passivated mixed-cation perovskite films for durable flexible perovskite solar cells with over 21% efficiency. *J. Mater. Chem. A*, **9**, 1574-1582 (2021).
14. Wu, S. et al. Low-bandgap organic bulk-heterojunction enabled efficient and flexible perovskite solar cells. *Adv. Mater.* **33**, 2105539 (2021).
15. Yang, L. et al. Record-efficiency flexible perovskite solar cells enabled by multifunctional organic ions interface passivation. *Adv. Mater.* **34,** 2201681 (2022).
16. Shao, Y. et al. Origin and elimination of photocurrent hysteresis by fullerene passivation in $CH_3NH_3PbI_3$ planar heterojunction solar cells. *Nat. Commun.* **5**, 5784 (2014).
17. Kumar, A. et al. A method for the non-destructive analysis of gradients of mechanical stresses by X-ray diffraction measurements at fixed penetration/information depths. *J. Appl. Cryst.* **39**, 633-646 (2006).
18. Delhez, R. et al. Role of x-ray diffraction analysis in surface engineering: Investigation of microstructure of nitrided iron and steels. *Surf. Eng.* **3**, 331-342 (1987).
19. Chen, Z. et al. Residual stress gradient analysis with GIXRD on ZrO_2 thin films deposited by MOCVD. *Surf. Coat. Technol.* **206**, 405-410 (2011).
20. Simeone, D. et al. Grazing incidence X-ray diffraction for the study of polycrystalline layers. *Thin Solid Films* **530**, 9-13 (2013).

21. Rolston, N. et al. Engineering stress in perovskite solar cells to improve stability. *Adv. Energy Mater.* **8**, 1802139 (2018).
22. Reyes-Martinez, M. et al. Time-dependent mechanical response of APbX3 (A = Cs, CH_3NH_3; X = I, Br) single crystals. *Adv. Mater.* **29**, 1606556 (2017).
23. Li, F. et al. Regulating surface termination for efficient inverted perovskite solar cells with greater than 23% efficiency. *J. Am. Chem. Soc.* **142**, 20134-20142 (2020).
24. Yang, Y. et al. Enhanced crystalline phase purity of $CH_3NH_3PbI_{3-x}Cl_x$ film for high-efficiency hysteresis-free perovskite solar cells. *ACS Appl. Mater. Interfaces* **9**, 23141-23151 (2017).
25. Xue, D. et al. Regulating strain in perovskite thin films through charge-transport layers. *Nat. Commun.* **11**, 1514 (2020).
