# Supplementary material for: Wearable perovskite solar cells by aligned liquid crystal elastomers
Source: Nat Commun. 2023 Mar 2;14:1204. doi: 10.1038/s41467-023-36938-7 (PMC9981560; doi:10.1038/s41467-023-36938-7)
Supplement: Supplementary file 2 — Solar Cells Reporting Summary [file 41467_2023_36938_MOESM2_ESM.pdf]

nature research

Corresponding author(s): Yanlin Song, Yiwang Chen, Meng Su, Lin Li

Solar Cells Reporting Summary

Nature Research wishes to improve the reproducibility of the work that we publish. This form is intended for publication with all accepted papers reporting the characterization of photovoltaic devices and provides structure for consistency and transparency in reporting. Some list items might not apply to an individual manuscript, but all fields must be completed for clarity.

For further information on Nature Research policies, including our data availability policy, see Authors & Referees.

Experimental design

Please check: are the following details reported in the manuscript?

1. Dimensions

Item	Reported	Details
Area of the tested solar cells	☒ Yes ☐ No	0.16 cm2. The information can be found in the Solar Cells characterizations part in Methods.
Method used to determine the device area	☒ Yes ☐ No	The perovskite solar cells were measured using a metal mask with the aperture area of 0.16 cm2. The information can be found in the Solar Cells characterizations part in Methods.

2. Current-voltage characterization

Item	Reported	Details
Current density-voltage (J-V) plots in both forward and backward direction	☒ Yes ☐ No	The J-V curves were scanned in the reverse direction unless specified otherwise. The J-V curves for the hysteresis study are obtained from scans in both forward and backward directions. The information can be found in the Solar Cells characterizations part in Methods and the plots are showed in Fig. 3b.
Voltage scan conditions<br>*For instance: scan direction, speed, dwell times*	☒ Yes ☐ No	All the J-V curves were measured under reverse scan with a scan rate of 0.2 V/s and a delay time of 30 ms unless otherwise specified. The information can be found in the Solar Cells characterizations part in Methods.
Test environment<br>*For instance: characterization temperature, in air or in glove box*	☒ Yes ☐ No	The J-V curves were measured in a nitrogen glovebox at room temperature. The information can be found in the Solar Cells characterizations part in Methods
Protocol for preconditioning of the device before its characterization	☒ Yes ☐ No	No preconditioning protocol was used before the characterization. The information is stated in the Solar Cells characterizations part in Methods.
Stability of the J-V characteristic<br>*Verified with time evolution of the maximum power point or with the photocurrent at maximum power point; see ref. 7 for details.*	☒ Yes ☐ No	Stabilized photocurrent output by holding the voltage at the maximum power point (1.0 V) for perovskite solar cells (Supplementary Fig. 18). The stability test information can be found in the Stability measurement part in Methods.

3. Hysteresis or any other unusual behaviour

Item	Reported	Details
Description of the unusual behaviour observed during the characterization	☒ Yes ☐ No	Small J-V hysteresis was observed in the optimized solar cells (Fig. 3b and Supplementary Fig. 18). The hysteresis study is discussed in the Performance and operational stability of PSCs in rigid versions part.
Related experimental data	☒ Yes ☐ No	We show the stabilized photocurrent output (Supplementary Fig. 18) and J-V plots under different directions in Fig. 3b.

4. Efficiency

Item	Reported	Details
External quantum efficiency (EQE) or incident photons to current efficiency (IPCE)	☒ Yes ☐ No	The EQE of the rigid device and the flexible device are shown in Supplementary Fig. 15 and Supplementary Fig. 25, respectively.
A comparison between the integrated response under the standard reference spectrum and the response measure under the simulator	☒ Yes ☐ No	The integrated current densities from the EQE curves are well-matched with the measured Jsc values. This is stated in the Performance and integration of flexible PSCs part.
For tandem solar cells, the bias illumination and bias voltage used for each subcell	☐ Yes ☒ No	There is no tandem cells in this work.

5. Calibration

Light source and reference cell or sensor used for the characterization	☒ Yes ☐ No	The J-V characteristics were measured by the Keithley 2400 source meter under simulated AM 1.5 sunlight at 100 mW cm-2 irradiance generated using an Enlitech solar simulator and the reference silicon solar cell was corrected from the National Renewable Energy Laboratories (NREL) to accurately estimate the equivalent AM 1.5 irradiance level. The information can be found in the Solar Cells characterizations part in Methods.
Confirmation that the reference cell was calibrated and certified	☒ Yes ☐ No	The reference silicon solar cell was certificated by NREL. The information can be found in the Solar Cells characterizations part in Methods.
Calculation of spectral mismatch between the reference cell and the devices under test	☒ Yes ☐ No	Before the measurement of each effective device, the intensity of the solar simulator was automatically detected by using the above reference silicon solar cells to calculate the precise power conversion efficiency. The information can be found in the Solar Cells characterizations part in Methods.

6. Mask/aperture

Size of the mask/aperture used during testing	☒ Yes ☐ No	The perovskite solar cells were measured using a metal mask with the aperture area of 0.16 cm2. The information can be found in the Solar Cells characterizations part in Methods.
Variation of the measured short-circuit current density with the mask/aperture area	☐ Yes ☒ No	The area of both rigid devices and flexible devices was corrected by calibrated apertures of 0.16 cm2.

7. Performance certification

Identity of the independent certification laboratory that confirmed the photovoltaic performance	☐ Yes ☒ No	This work mainly focuses on the efficiency loss from the rigid substrate to flexible substrate and the entire operational stability of PSCs.
A copy of any certificate(s) *Provide in Supplementary Information*	☐ Yes ☒ No	This work mainly focuses on the efficiency loss from the rigid substrate to flexible substrate and the entire operational stability of PSCs.

8. Statistics

Number of solar cells tested	☒ Yes ☐ No	50 devices.
Statistical analysis of the device performance	☒ Yes ☐ No	Statistical analysis of the performance for 50 devices are provided in Fig. 3c.

9. Long-term stability analysis

Type of analysis, bias conditions and environmental conditions *For instance: illumination type, temperature, atmosphere humidity, encapsulation method, preconditioning temperature*	☒ Yes ☐ No	See the Stability measurement in Methods.
